# Colorectal cancer in the Linxian China Nutrition Intervention Trial: Risk factors and intervention results

**DOI:** 10.1371/journal.pone.0255322

**Published:** 2021-09-15

**Authors:** Havva Keskin, Shao-Ming Wang, Arash Etemadi, Jin-Hu Fan, Sanford M. Dawsey, Christian C. Abnet, You-Lin Qiao, Philip R. Taylor

**Affiliations:** 1 Department of Internal Medicine, Goztepe Training and Research Hospital, Istanbul Medeniyet University, Istanbul, Turkey; 2 Department of Cancer Epidemiology, National Cancer Center/National Clinical Research Center for Cancer/Cancer Hospital, Chinese Academy of Medical Sciences and Peking Union Medical College, Beijing, China; 3 Metabolic Epidemiology Branch, Division of Cancer Epidemiology and Genetics, National Cancer Institute, National Institutes of Health, Bethesda, MD, United States of America; 4 Cancer Institute, Chinese Academy of Medical Sciences, Beijing, PR China; Hong Kong Polytechnic University, HONG KONG

## Abstract

**Background:**

Colorectal cancer (CRC) is among the most common cancers in economically developed countries and developing world. While dietary factors are associated with risk of CRC in the West and urban China, little is known about risk or protective factors in rural China.

**Methods:**

The Linxian General Population Nutrition Intervention Trial (NIT) cohort was established over 30 years ago to test whether daily multivitamin/mineral supplements could reduce the incidence and mortality of esophageal/gastric cardia cancer. The cohort included a total of 29,553 healthy participants 40–69 years old who were randomly assigned to supplements or placebos via a 2^4^ fractional factorial study design. We examined risk factors for the development of CRC as well as the effects of four different nutritional factors (Factor A: retinol, zinc; B: riboflavin, niacin; C: ascorbic acid, molybdenum; D: selenium, alpha-tocopherol, beta-carotene,) on CRC incidence following 5.25 years of supplementation in this randomized, placebo-controlled intervention trial.

**Results:**

CRC risk increased with age and height as well as piped water usage, family history of CRC, and consumption of foods cooked in oil, eggs, and fresh fruits. No effect on CRC was seen for any of these four intervention factors tested in both genders, but CRC was reduced 37% in females who received Factor D (selenium/alpha-tocopherol/beta-carotene) (RR = 0.63, 95% CI = 0.43–0.92, P = 0.016) compared to females who did not receive Factor D.

**Conclusions:**

In this undernourished rural Chinese population, CRC risk factors in this Chinese cohort showed both similarities and differences compared to Western and urban Asian Chinese populations. Intervention results suggested a potential benefit for women supplemented with selenium/alpha-tocopherol/beta-carotene.

## Introduction

Colorectal cancer (CRC) is the third most common cancer in men and the second in women worldwide [1]. More than half of CRC occur in more developed regions and incidence and mortality rates are highest among high-income countries; however, the number of cases has been rising recently in many low- and middle-income countries [1, 2]. There is wide geographical variability in the incidence of CRC worldwide where rates vary by up to ten-fold in both sexes [1]. In Asia, CRC rates have increased in China, the Philippines, and Thailand; decreased in Japan; and remained unchanged in India [1]. In 2012, CRC was the fifth most common malignancy among Chinese men and fourth in women, with age-standardized rates of 16.9 for men and 11.6 for women per 100,000 [1].

Similar to other cancers, age is a major risk factor for CRC, with 90% of all CRC cases diagnosed after age 50. In addition to male gender and African American race, other established CRC risk factors include smoking, alcoholic beverage use, high consumption of red and processed meat, body fatness, high adult attained height, family history of CRC, a personal history of inflammatory bowel disease, limited physical activity, and low intake of dietary fiber and calcium [3, 4]. Although there is increasing evidence that fruit and vegetables protect against some cancers [5], the precise role of fruit, vegetables, and other dietary factors in the development of CRC is inconclusive [6, 7].

The increasing rate of CRC in China is almost certainly linked to changes in socioeconomic status leading to a more Western lifestyle and modified dietary patterns. According to the International Agency for Research on Cancer (IARC), increased obesity in China is related to CRC [8]. Several prospective studies about risk factors of CRC have been published in Asian Chinese, but except for one [9], these studies were limited to urban Chinese populations in Shanghai and Singapore where CRC risk from exposures to body fatness, tobacco, and alcohol mostly show results similar to Western cohorts [10–15], while the role of dietary factors is more complicated and variable [16, 17].

Multivitamin and mineral usage has increased in recent decades, especially in Western countries where use is motivated in large part to prevent cancer, other chronic diseases, and death. Randomized trials testing vitamin/mineral supplement effects on total mortality have produced variable results, including increased mortality [18–23], no effect [19, 24, 25], and decreased mortality [19, 26–28].

The General Population Nutrition Intervention Trial (NIT) was a cancer prevention trial conducted in Linxian, China where rates of esophageal cancer are the highest in China and the world. The NIT tested whether daily multivitamin/mineral supplements could reduce the incidence and mortality of esophageal/gastric cardia cancer among 29,553 adults supplemented for 5.25 years between 1986 and 1991. In the current analysis, we evaluated the risk factors for the development of CRC in the NIT cohort which has now been followed for 30 years. Since the NIT was a randomized controlled trial, we also examined the effects of the interventions on the incidence of CRC over 30 years in this rural Chinese population.

## Materials and methods

**Fig 1:**
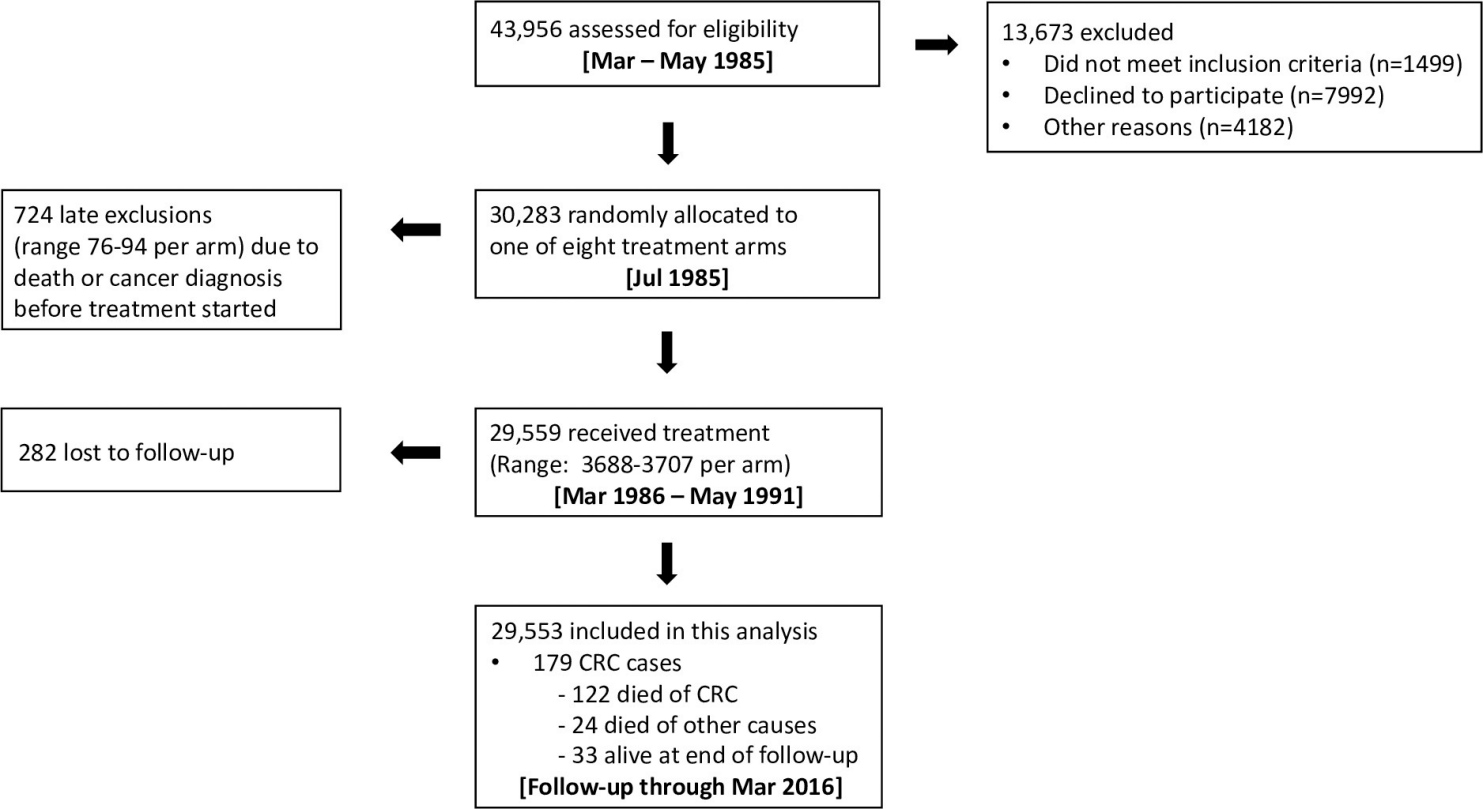
CONSORT trial flow diagram of the Linxian general population trial.

The design, interventions, methods (including eligibility criteria and exclusions), and endpoints for the trial were described in detail previously [28–30] and are summarized in Fig 1, CONSORT trial flow diagram. In brief, the participants were 29,553 healthy adults 40–69 years old from four Linxian communes who were interviewed in the spring of 1985 and given a brief physical examination which included measured height and weight. A baseline questionnaire captured demographic and lifestyle characteristics (eg, age, birthplace, education, occupation, diet, tobacco and alcohol use, water supply, personal and family history of cancer, and personal history of selected symptoms and conditions) [28–30].

Participants were randomly assigned to supplements or placebos from March 1986 through May 1991 (5.25yr) according to a 2^4^ fractional factorial study design as previously described [28–30]. The four factors tested included factor A (5000 IU vitamin A and 22.5 mg zinc oxide), factor B (3.2 mg riboflavin and 40 mg niacin), factor C (120 mg ascorbic acid and 30 ug molybdenum), and factor D (50 ug selenium, 30 mg alpha-tocopherol, and 15 mg beta-carotene). Doses for these daily supplements ranged from one to two times the United States Recommended Daily Allowances [30]. Written informed consent was obtained from each participant before trial enrollment, and human subject protection procedures were followed in accordance to those prescribed by the U.S. National Institutes of Health and the Chinese Academy of Medical Sciences.

The CONSORT trial flow diagram of the NIT cohort is seen in Fig 1. During the trial period, village health workers visited participants monthly to deliver pills, assess pill compliance, and ascertain vital and disease status. Diagnostic materials (case records, pathology slides, and/or x-rays) for 85% of all cancer cases in the trial period were available and were reviewed by a panel of American and Chinese experts. Monthly checks by village doctors, and quarterly contact with the local commune and county hospitals were used to ascertain new cancer diagnoses and causes and fact of death throughout the follow-up period [28, 29]. Information on CRC diagnosis, including anatomic site (colon or rectum), was available for all 179 incident CRC cases analyzed here through the Linxian Cancer Registry and/or various diagnostic modalities. Diagnosis was based on pathology (histology or cytology) for 93 (52%) cases, x-ray for 36 (20%) cases, surgical or endoscopic procedure/visualization for 25 (14%) cases, and clinical or other records for 25 (14%) cases.

The protocol for this study was approved by the Institutional Review Boards of both the National Cancer Institute, National Institutes of Health, Bethesda, MD, USA. Also, the protocol for this study was approved by the Cancer Institute, Chinese Academy of Medical Sciences, Beijing, PR China. All patients provided written informed consent prior to enrolling in this study according to institutional guidelines. The study was performed in accordance with the Declaration of Helsinki. **Clinical trial registration:** ClinicalTrials.gov Identifier: NCT00342654

### Statistical analysis

Follow-up started with the beginning of the intervention in March 1986 and continued until date of cancer diagnosis, date of death, or the end of the follow-up period (March 2016), whichever came first. Descriptive statistics were used to summarize exposures and outcomes. We used Cox proportional hazard regression models with maximum partial likelihoods and partial score functions to estimate hazard ratios and corresponding 95% confidence intervals (95% CIs), adjusted for potential confounders. In order to identify potential risk factors for CRC in this population, we’ve interpreted these hazard ratios as relative risks (RRs) throughout this paper. Variables adjusted for in analyses for each table are described in the footnotes. The results are presented for the total cohort and by gender.

Body mass index (BMI), height, and weight were divided into category-specific tertiles for the total cohort and by gender. We also examined and include here China-specific BMI categories. Ever smoker (cigarette or pipe) was categorized as yes/no according to whether a person smoked regularly for six months or more. Current smoker (cigarette or pipe) was defined as those who smoked regularly at the time of the interview. The amount of tobacco used by pipe smokers was converted to cigarette equivalents (0.8 g tobacco per cigarette). Intensity and duration analyses included all former and current smokers. Alcohol intake was based on reported intake in the previous 12 months. Food consumption evaluated in the baseline questionnaire included frequency of intake over the past year for food cooked in oil, meat (pork, beef, rabbit, chicken, or duck), eggs, fresh fruit, fresh vegetables, dried fruit, dried vegetables, pickled vegetables, moldy vegetables, moldy bread, and millet chaff/persimmon bread. Consumption frequencies of food items were converted into frequency per year (servings/year) and categorized into category-specific tertiles as described for BMI, height, and weight. Relative risks (RRs) for cancer were calculated with the lowest category for BMI, height, weight, education or the lowest consumption category for food as the referent. Tests for trend were performed by assigning a single ordinal variable to each category evaluated. All P values were from two-sided Wald tests. P values less than 0.05 were considered nominally statistically significant. Only relations with P<0.05 were referred to as “associations” or “associated”. All analyses were implemented in IBM SPSS Statistics for Windows, Version 25.0., Armonk, NY:IBM Corporation.

For the analysis of intervention results, separate Cox proportional hazards regression models were used to estimate RRs and 95% CIs for All, Males, and Females. The model for All participants included adjustment for the three factors that trial participants were blocked on at randomization (commune, age, and gender) and four separate indicator variables which represented each of the four intervention treatment groups evaluated in the model (Factor A = 0 if not A, 1 if A; Factor B = 0 if not B, 1 if B; Factor C = 0 if not C, 1 if C; and Factor D = 0 if not D, 1 if D). Cox models for Males and Females were identical to the model for All except that no adjustment for gender was done.

## Results

During the total 30-year follow-up, 179 incident CRC cases, including 63 colon and 116 rectum, were diagnosed in the entire cohort. The mean age of the cohort at the start of follow-up was 52 years (52.6 for 13,175 males and 51.4 for 16,378 females). Distributions of demographic and lifestyle factors for the total cohort and by gender are presented in **S1 Table in S2 File** As shown in the table, tobacco and alcohol use were predominantly male habits (67% of men vs 0.2% of women ever used tobacco, and 40% of men vs 10% of women consumed alcohol in the previous year).

Risk associations for age, gender, anthropometric variables, socioeconomic status factors (education, water piped into the home), smoking, alcohol intake, family history of CRC or any cancer, and birth place are shown in **Table 1**. Age was a risk factor for CRC in the total cohort and in both males and females. Of the anthropometric variables examined, in both the total cohort and in females, risk for increased BMI in Chinese-specific categories trended higher and increased height was strongly associated with increased risk, while BMI in tertiles and weight were not associated with risk in any group. Persons who had water piped into their home had a 51% increased risk of CRC compared to those whose water came from other sources. Although a family history of CRC was uncommon in this cohort (93 persons or 0.3% of cohort; **S1 Table in S2 File**) and in the CRC cases (2 cases, both males, or 3% of male cases), males with a positive family history had an eight-fold increased risk of CRC (**Table 1**, footnote^f^). There were no associations between CRC and gender, level of education, being born in Linxian, any alcohol usage in the previous year, ever use of tobacco, or family history of any cancer.

**Table 1:** Multivariate-adjusted RRs and 95% CIs for colorectal cancer for selected characteristics/exposures in the total cohort and by gender^a^.

	Total Cohort		Gender Group			
	(n = 29,553)					
Characteristic/exposure			Male		Female	
			(n = 13,175)		(n = 16,378)	
	No. CRC	RR (95% CI; p-value)	No. CRC	RR (95% CI; p-value)	No. CRC	RR (95% CI; p-value)
**Age (year)** ^**b**^	179	**1.03 (1.01–1.05;0.004)**	67	**1.04 (1.00–1.07;0.034)**	112	**1.03 (1.00–1.06;0.033)**
**Gender(male)** ^**b**^	67	0.77 (0.45–1.31)		-		-
**BMI (kg/m**^**2**^**)**^**b**^^**,**^^**c**^ • T_1_ • T_2_ • T_3_	55 53 71	1 0.92 (0.63–1.34) 1.18 (0.83–1.69) P_Trend_ = 0.285	23 18 26	1 0.78 (0.44–1.38) 0.88 (0.48–1.60) P_Trend_ = 0.699	31 34 47	1 1.02 (0.62–1.69) 1.39 (0.89–2.19) P_Trend_ = 0.114
**BMI (kg/m**^**2**^**)**^**b**^^**,Chinese**^ • <18.50 • 18.50–23.99 • ≥24.0	6 139 34	1 1.60 (0.70–3.64) 1.68 (0.70–4.01) **P**_**Trend**_ **= 0.017**	2 58 7	1 1.09 (0.27–4.48) 0.90 (0.19–4.39) P_Trend_ = 0.622	4 81 27	1 1.83 (0.67–5.00) 2.06 (0.72–5.91) **P**_**Trend**_ **= 0.002**
**Height (m)**^**b**^^**.**^^**c**^ • T_1_ • T_2_ • T_3_	49 75 55	1 **1.63 (1.12–2.37;0.011)** **1.80 (1.08–3.01;0.025)** **P**_**Trend**_**= 0.016**	15 28 24	1 1.57 (0.37–6.77) 1.72 (0.41–7.12) P_Trend_ = 0.495	28 33 51	1 **1.61 (1.08–2.38;0.018)** 1.83 (0.89–3.78) **P**_**Trend**_ **= 0.014**
**Weight (kg)**^**b**^^**,**^^**c**^ • T_1_ • T_2_ • T_3_	50 67 62	1 1.39 (0.95–2.03) 1.41 (0.93–2.12) P_Trend_ = 0.116	21 20 26	1 1.33 (0.55–3.24) 1.26 (0.52–3.03) P_Trend_ = 0.783	27 42 43	1 1.35 (0.88–2.08) 1.48 (0.91–2.41) P_Trend_ = 0.090
**Education**^**b**^ • No formal education • 1–5 year • >5 years	79 70 30	1 0.81 (0.55–1.19) 0.71 (0.44–1.13) P_trend_ = 0.139	15 37 15	1 0.63 (0.34–1.17) 0.65 (0.29–1.45) P_trend_ = 0.317	64 33 15	1 0.94 (0.56–1.56) 0.70 (0.39–1.26) P_trend_ = 0.252
**Born in Linxian (yes)** ^**b**^	172	1.13 (0.53–2.41)	64	0.73 (0.23–2.35)	108	1.44 (0.53–3.90)
**Water piped into home (yes)** ^**b**^	60	**1.50 (1.10–2.05;0.011)**	26	**1.94 (1.18–3.18;0.009)**	34	1.27 (0.85–1.90)
**Alcohol intake (yes)** ^**b**^ ^**,**^ ^**d**^	46	1.24 (0.85–1.79)	30	1.17 (0.71–1.91)	16	1.36 (0.79–2.31)
**Ever smoke (yes)** ^**b**^ ^**,**^ ^**e**^	41	0.79 (0.48–1.30)	41	0.82 (0.49–1.35)	0	-
**Family history of any cancer (yes)** ^**b**^ ^**,**^ ^**f**^	59	0.96 (0.70–1.31)	21	0.87 (0.52–1.47)	38	1.02 (0.69–1.51)

^a^Multivariate-adjusted Cox proportional hazards regression models were used to estimate all RRs and 95% CIs shown. P-values are provided for all RRs whenever the p-value is less than 0.05 and for all P_trend_ tests. Bolded RRs and P_trend_ tests indicate P<0.05.

^**b**^**For the total cohort**: RRs and 95% CIs shown for the total cohort above came from a single multivariate model that included all 12 characteristic/exposure variables (only one BMI variable was used at a time). Body size variables were coded as BMI (divided into tertiles as T1,T2,T3 represented by indicator variables) or BMI Chinese(divided into China-specific categories as <18.50, 18.50–23.99, ≥24.0 represented by indicator variables); Height (divided into tertiles as T1,T2,T3 represented by indicator variables); and Weight (divided into tertiles as T1,T2,T3 represented by indicator variables) where the first tertile was the reference category for each variable.

Non-body size variables included in the multivariate model were Age (continuous); Gender (0 = F,1 = M); Education (0,1,2 where 0 = No formal education, 1 = 1–5 years, and 2 = >5 years); Born in Linxian (0 = no,1 = yes); Water piped into home (0 = no,1 = yes); Alcohol intake (0 = no,1 = yes); Ever smoke (0 = no,1 = yes); and Family history of any cancer (0 = no,1 = yes). P_trend_ tests (for the ordinal variables of BMI, BMIChinese, Height, Weight, and Education) were evaluated as a single ordinal variable (0,1,2) to account for the three categories of each variable as shown and ordered in the table. **For Gender Groups:** RRs and 95% CIs shown for Male and Female categories separately above came from separate multivariate models for each gender that included the same 12 characteristic variables described above for the total cohort except no Gender variable was included.

**Further description of variables**:

^c^Body size variables: (BMI, height, weight) were divided into category-specific tertiles as follows: For the total cohort: BMI (kg/m)<20.81, 20.81–22.66, >22.66; Height (m) <1.55, 1.55–1.62, >1.62; Weight (kg) <51.50, 51.50–58, >58. For males: BMI <20.46, 20.46–22.34, >22.34; Height <1.62, 1.62–1.67, >1.67; Weight <55, 55–61, >61. For females: BMI <20.81, 20.81–22.94, >22.94; Height <1.52, 1.52–1.55, >1.55; Weight <49, 49–55, >55. Non-body size variables:

^d^Alcohol intake was evaluated as any in previous 12 months.

^e^Ever smokers were defined as those who had ever smoked regularly for at least 6 months.

^f^Family history of any cancer is in 1st-degree relative(s). There was a positive Family history of CRC in only 0.3% of the total cohort, distributed as 2 in CRC cases and 91 in non-case cohort members. Both cases were in Males where a total of 45 Males reported a positive FH of CRC (2 in cases, 43 in non-case cohort). The RRs (95% CI) for FH of CRC were, therefore, 3.36 (0.82–13.80) in the total cohort and 9.53 (2.20–41.16) in the Males.

Detailed analyses of CRC risk associated with tobacco exposure are shown in **Table 2**. Since <1% of women ever smoked, these analyses were limited to men only. No risk associations for cigarettes or pipe use, by ever use, duration or intensity of use, or cumulative exposure (as pack-years of cigarettes or cigarette equivalents) were seen.

**Table 2:** Age-adjusted RRs and 95% CIs for colorectal cancer in men by baseline smoking characteristics^a^.

Smoking characteristic	Total	Colorectal	Colorectal
	Cohort %	Cancer %	Cancer RR
	(n = 13,175)	(n = 67)	(95% CI)
**Ever smoke**^**b**^ • No smoke • Cigarette smoking only • Cigarette+Pipe smoking^x^	32.6 49.1 17.9	38.8 (26) 44.8 (30) 16.4 (11)	1 0.78 (0.46–1.32) 1.00 (0.49–2.07)
**Cigarette pack-years** • No smoke • <15.75 • ≥15.75	33.4 33.3 33.3	38.8 (26) 34.3 (23) 26.9 (18)	1 0.92 (0.52–1.62) 0.79 (0.43–1.44) P_Trend_ = 0.43
**Cigarette duration (years)**^**c**^ • No smoke • <28 • ≥28	33.4 33.1 33.5	38.8 (26) 35.8 (24) 25.4 (17)	1 1.07 (0.55–2.08) 0.99 (0.40–2.44) P_Trend_ = 0.98
**Cigarette intensity (cigarettes/day)**^**c**^ • No smoke • <11 • ≥11	33.2 36.0 31.8	38.8 (26) 35.8 (24) 25.4 (17)	1 1.04 (0.54–2.00) 1.08 (0.35–3.38) P_Trend_ = 0.89
**Pipe pack-years (as cigarette equivalent)** • No smoke • <2.67 • ≥2.67	81.8 9.0 9.2	83.6 (56) 11.9 (8) 4.5 (3)	1 1.41 (0.63–3.13) 0.69 (0.21–2.24) P_Trend_ = 0.54
**Pipe duration (years)**^**d**^ • No smoke • <11 • ≥11	81.8 9.0 9.2	83.6 (56) 11.9 (8) 4.5 (3)	1 1.51 (0.70–3.27) 0.44 (0.09–2.84) P_Trend_ = 0.484
**Pipe intensity (as cigarette equivalents/day)**^**d**^ • No smoke • <5 • ≥5	81.8 8.6 9.6	83.6 (56) 9.0 (6) 7.5 (5)	1 1.83 (0.63–4.46) 0.74 (0.47–4.92) P_Trend_ = 0.919
**Tobacco pack-years (as cigarettes and pipe use, cigarette equivalents combined)**^**e**^ • No smoke • ≤16 • >16	33.0 33.3 33.7	38.8 (26) 32.8 (22) 28.4 (19)	1 0.86 (0.49–1.53) 0.82 (0.45–1.49) P_Trend_ = 0.54
**Tobacco duration (years**)^f^ • No smoke • <29 • ≥29	33.2 33.7 33.1	38.8 (26) 38.8 (26) 22.4 (15)	1 1.08 (0.56–2.06) 0.78 (0.31–1.97) P_Trend_ = 0.727
**Tobacco intensity (as cigarettes and pipe use, cigarette equivalents combined/day)**^**f**^ • No smoke • <14 • ≥14	32.9 31.6 35.6	38.8 (26) 31.3 (21) 29.9 (20)	1 0.99 (0.52–1.87) 1.11 (0.40–3.14) P_Trend_ = 0.83

^a^Separate age-adjusted Cox proportional hazards regression models were used to estimate RRs and 95% CIs for each of the 10 different smoking characteristic analyses shown here. As well, separate age-adjusted Cox models were also used to perform P_trend_ tests for each of the 9 different smoking characteristics in which this test was conducted (no P_trend_ test was performed for the Ever smoke variable). P_trend_ tests were evaluated as a single ordinal variable (coded 0,1,2) to account for the three categories of each variable as shown and ordered in the table.

^b^Ever smoke (cigarette or pipe) was defined as a person who ever smoked regularly for at least 6 months.

^c^In addition to the age, this model also adjusted for cigarette pack-years.

^d^In addition to the age, this model also adjusted for pipe pack-years.

^e^Cigarette and pipe combined as cigarette equivalents.

^f^In addition to the age, this model also adjusted for tobacco pack-years.

Distributions of food intake and cooking practices are shown in **S2 Table in S2 File,** while associations between intake of foods and CRC risk are found in **Table 3**. Associations with risk were seen for four foods or cooking practices. More frequent consumption of food cooked in oil was associated with increased CRC risk in the total cohort as well as in females. CRC risk increased in persons with the higher intake of meat and eggs (in the total cohort and in males) and fresh fruit (in males). Moldy vegetable consumption was reported in only 1% of the total cohort population, and no CRC cases reported this habit. No evidence of association with risk of CRC was seen either in the total cohort or separately in males or females with consumption of persimmon bread, fresh vegetables, dried vegetables or fruit, pickled vegetables, or moldy bread.

**Table 3:** Multivariate-adjusted RRs and 95%CIs for colorectal cancer by consumption of selected foods in the total cohort and by gender^a^.

Consumption frequency	Total		Gender Group			
	Cohort		Male		Female	
(Times/year)	(n = 29,553)					
			(n = 13,175)		(n = 16,378)	
	No. CRC	RR (95% CI; p-value)	No. CRC	RR (95% CI;p-value)	No. CRC	RR (95% CI;p-value)
**Foods cooked in oil**^**b**^^**,**^^**c**^ • T_1_ • T_2_ • T_3_	40 75 64	1 **1.60 (1.08–2.35;0.018)** **1.57 (1.05–2.34;0.027)** P_Trend_ = 0.151	25 19 23	1 1.99 (1.00–3.95;0.050) 1.42 (0.69–2.94) P_Trend_ = 0.891	29 42 41	1 1.40 (0.87–2.25) **1.70 (1.05–2.75;0.029)** P_Trend_ = 0.051
**Meat**^**b**^^**,**^^**c**^ • T_1_ • T_2_ • T_3_	54 88 37	1 1.01(0.72–1.42) 1.51 (0.97–2.35) **P**_**Trend**_ **= 0.040**	16 28 23	1 0.90 (0.47–1.68) 1.61 (0.81–3.20) **P**_**Trend**_ **= 0.046**	37 32 43	1 1.06 (0.71–1.60) 1.33 (0.72–2.47) P_Trend_ = 0.355
**Eggs**^**b**^^**,**^^**c**^ • T_1_ • T_2_ • T_3_	41 75 63	1 1.10 (0.75–1.61) **1.65 (1.10–2.46;0.015)** **P**_**Trend**_ **= 0.005**	16 20 31	1 1.07 (0.52–2.19) **2.14 (1.06–4.32;0.034)** **P**_**Trend**_ **= 0.004**	30 40 42	1 1.13 (0.71–1.78) 1.37 (0.83–2.27) P_Trend_ = 0.241
**Fresh fruits**^**b**^^**,**^^**c**^ • T_1_ • T_2_ • T_3_	57 43 79	1 0.98 (0.68–1.42) 1.36 (0.95–1.95) P_Trend_ = 0.051	14 15 38	1 0.93 (0.47–1.84) **1.97 (1.08–3.59;0.027)** **P**_**Trend**_ **= 0.004**	37 42 33	1 1.02 (0.66–1.58) 1.05 (0.65–1.68) P_Trend_ = 0.869
**Fresh vegetables**^**b**^^**,**^^**c**^ • T_1_ • T_2_ • T_3_	56 48 75	1 0.97 (0.66–1.43) 1.01 (0.60–1.71) P_Trend_ = 0.999	20 20 27	1 1.17 (0.63–2.19) 1.05 (0.46–2.41) P_Trend_ = 0.834	35 29 48	1 0.86 (0.52–1.41) 0.96 (0.49–1.91) P_Trend_ = 0.796
**Pickled vegetables**^**b**^ • 0 • ≥1	163 16	1 0.92 (0.55–1.54)	66 1	1 0.19 (0.03–1.34)	97 15	1 1.27 (0.73–2.19)
**Millet chaff/ Persimmon bread**^**b**^ • 0 • ≥1	172 7	1 0.92 (0.43–1.96)	65 2	1 0.75 (0.18–3.07)	107 5	1 1.00 (0.41–2.47)
**Moldy bread**^**b**^ • 0 • ≥1	149 30	1 0.92 (0.62–1.36)	58 9	1 0.88 (0.43–1.79)	91 21	1 0.91 (0.57–1.47)

^a^Separate multivariate-adjusted Cox proportional hazards regression models were used to estimate RRs and 95% CIs for each of the 9 different analyses shown here. Foods cooked in oil, Meat, Eggs, Fresh fruits, and Fresh vegetables were divided into tertiles as T1,T2,T3 represented by indicator variables with the first tertile serving as the reference category for each variable. P_trend_ tests for these ordinal variables were evaluated as a single ordinal variable (0,1,2) to account for the three categories of each variable as shown and ordered in the table. P-values are provided for all RRs whenever the p-value is less than 0.05 and for all P_trend_ tests. Bolded RRs and P_trend_ tests indicate P<0.05.

^b^Multivariate models were adjusted for: Age (continuous), Gender (0,1), BMI (continuous), Education (0,1,2), Born in Linxian (0 = No,1 = Yes), Water piped into home (0 = No,1 = Yes), Alcohol intake (0 = No, 1 = Yes), Smoking (0 = No, 1 = Yes), and Family history of any cancer (0 = No, 1 = Yes). For more details regarding coding of these variables, see footnotes to Table 1.

^c^Further description of consumption frequency variables (times/year) divided into tertiles in the table: Foods cooked in oil, meat, eggs, fresh vegetables, and fresh fruits consumption were divided into category-specific tertiles as follows: **For total cohort:** Foods cooked in oil <8, 8–12, >12; meat <6, 6–12, >12; eggs <4, 4–24, >24; fresh fruits <6, 6–23, >23; fresh vegetables <730, 730–912, >912. **For males:** Foods cooked in oil <10, 10–20, >20; meat <8, 8–12, >12; eggs <5, 5–24, >24.00; fresh fruits <5, 5–16, >16; fresh vegetables <549, 549–731, >732. **For females:** Foods cooked in oil <8, 8–12, >12; meat <5, 5–10, >10; eggs <4, 4–12, >12; fresh fruits <4, 4–17, >17; fresh vegetables <549, 549–730, >730.

Separate RRs and 95% CIs for colon and rectal cancer risks are shown in **S3 Table in S2 File** for selected characteristics/exposures for consumption of selected foods in **S4 Table in S2 File**, and colon and by intervention factors in **S5 Table in S2 File**. In general, results for colon and rectum were similar to CRC overall. As well, risks for colon were generally similar to rectum. Nominally significant results for colon included age, height, and egg and fresh fruit consumption, while nominally significant findings for rectum included BMI, water piped into home, and foods cooked in oil. There were no significant intervention effects in either colon or rectum.

When the main effects of the four different intervention factors were assessed in the total cohort, no associations were found (**Table 4**). However, subgroup analyses showed that CRC was reduced by 37% in females who received Factor D (selenium/alpha-tocopherol/beta-carotene) (RR = 0.63, 95% CI = 0.43–0.92, P = 0.016) compared to females who did not get Factor D.

**Table 4:** RRs and 95% CIs for colorectal cancer by intervention group in all trial participants and by gender.

	All (n = 179 CRCs)^a^		Males (n = 67 CRCs)^b^		Females (n = 112 CRCs)^b^	
	RR (95% CI)	P	RR (95% CI)	P	RR (95% CI)	P
**Factor A** ^**c**^	1.07 (0.80–1.43)	0.659	0.73 (0.45–1.18)	0.200	1.33 (0.91–1.93)	0.137
**Factor B** ^**c**^	0.79 (0.59–1.06)	0.118	0.90 (0.56–1.46)	0.680	0.74 (0.51–1.07)	0.111
**Factor C** ^**c**^	1.07 (0.80–1.43)	0.641	0.66 (0.41–1.08)	0.096	1.42 (0.97–2.07)	0.070
**Factor D** ^**c**^	0.77 (0.57–1.03)	0.081	1.09 (0.68–1.76)	0.724	**0.63 (0.43–0.92)**	**0.016**

^a^Separate multivariate Cox proportional hazards regression models were used to estimate RRs and 95% CIs for All, Males, and Females. The model for All participants included adjustment for the 3 factors that trial participants were blocked on at randomization (ie, commune, age, and gender) and 4 separate indicator variables which represented each of the 4 intervention treatment groups evaluated in the model (Factor A = 0 if not A, 1 if A; Factor B = 0 if not B, 1 if B; Factor C = 0 if not C, 1 if C; and Factor D = 0 if not D, 1 if D).

^b^The Cox models for Males and Females were identical to the model for All except that no adjustment for gender was done.

^c^Micronutrients included in the 4 intervention treatments were: Factor A (5000 IU vitamin A and 22.5 mg zinc oxide), factor B (3.2 mg riboflavin and 40 mg niacin), factor C (120 mg ascorbic acid and 30 ug molybdenum), and factor D (50 ug selenium, 30 mg alpha-tocopherol, and 15 mg beta-carotene).

## Discussion

We evaluated the potential role of demographic and lifestyle factors as well as consumption of various foods as risk or protective factors for CRC in rural China. We studied these factors in the context of a cohort initially established as a randomized, controlled cancer prevention trial to test the role of multivitamin/mineral intake in the prevention of upper GI cancers. After 30 years of follow-up, the 179 new CRCs identified in this cohort represent the most comprehensive evaluation of CRC risk in rural China to date. Our study found increased risk of CRC with a number of factors, including age and height as well as use of piped water in the home, and increased consumption of four three items, namely, cooking oil, eggs, and fresh fruit. Significant trend tests also suggested risk associations for increased BMI (Chinese categories only) and increased meat consumption, although relative risks for point estimates within categories were not themselves significant. Our analysis of the cohort as a randomized trial hinted at a protective effect for CRC in the entire trial in persons randomized to the group that received pills containing the combination of selenium, alpha-tocopherol, and beta-carotene (RR = 0.77, P = 0.08). That suggestive protective effect in the entire population was nominally significant in females (RR = 0.63, P = 0.016) but not in males (RR = 1.09, P = 0.72).

While there are numerous established risk factors for CRC derived from studies in Western populations [3, 4], only a few prospective cohort studies of CRC risk factors have been conducted in Asian Chinese, and all but one of these have been in urban populations [11, 12]. Studies of CRC in Asian Chinese have emerged only recently as rising rates have increased interest in etiology and prevention. However, only a limited number of Western risk factors have been examined in these studies. In the present study, we compared information on Western risk factors available to us from the baseline questionnaire of a prospective cohort study established in rural China in 1985 with published results for CRC risk factors from other prospective cohort studies conducted in Asian Chinese, including one other rural cohort study in Jiashan County [9, 31, 32] and four cohort studies from urban areas (Shanghai Mens’ Health Study-SMHS-[11, 14, 16], Shanghai Womens’ Health Study -SWHS-[11, 33], Singapore Health Study [12, 13, 17], and Shanghai Cohort Study-SCS-[15, 34]); see summary comparison of CRC risk factors in these prospective cohort studies of Asian Chinese in **S6 Table in S2 File**. We also report selected associations with some exposures that have not been established as risk factors for CRC in Western studies.

The NIT cohort had available information on four non-diet risk factors, including age, gender, smoking, and family history of CRC. Of these four factors, only increasing age (in both males and females) and a positive family history (in males only) were associated with increased risk, although the family history association was based on small numbers (only 0.3% of the cohort and two cases had a positive history). Age was also a CRC risk factor in the SWHS; no data were reported for age in the Singapore, SCS, or Jiashan cohorts. No association with family history of CRC was seen in the SWHS. The other Asian Chinese cohorts did not report on this. Smoking was positively related to CRC risk in both the Singapore and SMHS cohorts, but not the SCS or Jiashan cohorts. Gender associations were not reported in the other Asian Chinese cohorts. In addition, data on body size (weight, height, BMI) were available in the NIT, and increased weight (women only) and height (both genders) were associated with risk of CRC, but BMI was not. While height was not reported on in the Asian Chinese cohorts, various measures of body fatness (BMI, waist-to-hip ratio, and/or waist circumference) were associated with increased CRC risk in several of these cohorts (**S6 Table in S2 File**) [9, 11, 12, 15].

Dietary constituents are among the most studied risk factors for CRC. Unfortunately, Western and Asian diets differ substantially, and questionnaire-based assessments of these diets in the studies available for comparison here have limited overlap and comparability. We found CRC risk increased in the NIT in association with increased consumption of four food items—red meat, eggs, total fruit, and foods cooked in oil. By comparison, red meat consumption was not related to CRC risk in either the rural or any of the urban Asian Chinese cohorts. Persons who ate more eggs were also at increased risk of CRC in the SWHS cohort. In contrast to our NIT finding of increased risk for eating fruit, SMHS fruit eaters had reduced risk of CRC, while the relation of fruit to CRC was null in the SWHS and SMHS but not reported in the SCS or Jiashan cohorts. Eating foods cooked in oil increased risk in the NIT, but not in the SWHS. Several other food items associated with risk in Western studies that showed no relation to risk in the NIT included: (i) alcohol consumption was not related to CRC risk in either rural Chinese cohort (NIT or Jiashan) but was associated with risk in two urban Chinese cohorts (Singapore and SMHS); and (ii) vegetable consumption was unassociated with risk in the NIT or the other five Asian Chinese cohorts compared here. Other diet associations have been reported (with both increased and decreased risks) from the Singapore, SMHS, and SWHS cohorts, but these food items were not assessed or reported in the NIT or Jiashan studies (**S6 Table in S2 File**) [9, 13, 14, 16, 17, 32–34].

There are only limited data on the role of water source on CRC risk from the cohort studies of Asian Chinese. Although the urban cohorts have not reported on this association, the rural cohorts examined water source and reported associations with increased risk with either use of water either piped into the home or from well water [9, 31]. Previously in the NIT, we considered piped water as a measure of high socioeconomic status as it, along with higher education level and increased consumption of meat, eggs, and fresh fruits, were all protective factors for esophageal cancer [35]. More work and thought are needed to determine if and why water source relates to CRC risk in rural China.

A comparison of the most established risk factors for CRC suggests that there is at least some evidence from both Western and Chinese cohort studies to support a role for increased age, a positive family history, tobacco use, increased body size, decreased physical activity, alcohol use, higher intake of processed and red meat, and lower intake of dairy as risk factors in both cultures (**S6 Table in S2 File**). However, data from China are limited and inconsistent, and large differences in diets between cultures complicate the direct comparison of diets, foods, or the intake of nutrients across such studies.

The strongest evidence we have from the current study for the role of diet in the prevention of CRC comes from our analysis of the intervention effects of micronutrients given as part of the NIT, a randomized, placebo-controlled trial (RCT). Our results showed a suggestive benefit for Factor D (selenium/alpha-tocopherol/beta-carotene) in all trial participants and a 37% reduction in CRC incidence in females. To our knowledge, this is among the first large RCT to test micronutrients in the prevention of CRC in China. There have, of course, been numerous RCTs in Western countries that have evaluated the potential effects of various micronutrients on the development of CRC or colorectal adenomas, the obligate precursors to CRC. We summarized nine such micronutrient trials to prevent colorectal adenomas (**S7 Table in S2 File**) and 14 micronutrient trials to prevent CRC (**S8 Table in S2 File**) in the Supplementary materials.

**S7 Table in S2 File** shows the nine micronutrient RCTs that evaluated the effects of seven different micronutrients (singly, in combination, or factorially) on the development of colorectal adenomas. Only calcium supplementation resulted in nominally significant effects. The initial report from the Calcium Polyp Prevention Trial found a 19% reduction in polyps in the calcium supplemented group [36], while the Calcium Follow-up Study, a 10 year post-intervention observation period of this same RCT, showed that the observed benefit was retained through the first five years of follow-up (37% reduction) but was no longer evident thereafter [37]. The potential benefit of calcium supplementation was further amplified by results from another pilot RCT which reported a 53% reduction in new polyps in persons who received calcium supplements [38]. The long-term benefit of calcium on polyp prevention was challenged, however, by findings from a third RCT, the Vitamin D/Calcium Polyp Prevention Trial, whose initial intervention results were null [39], but a post-intervention observation phase follow-up showed a 166% increase in a subgroup of polyps (sessile serrated adenomas or polyps) in persons randomized to calcium supplementation [40]. Separately, treatment effects for all polyps combined in the post-intervention observation phase were null [41]. None of the other six micronutrients tested in these nine RCTs (ie, folate, B6, B12, vitamin D, selenium, and alpha-tocopherol) showed either efficacy or harm (**S7 Table in S2 File**).

Fourteen RCTs that tested 10 different micronutrients and a multivitamin for the prevention of CRC are identified in **S8 Table in S2 File** (only trials with at least 25 total CRCs that reported results by treatment group assignment are shown). Few if any of these RCTs were conducted specifically to evaluate the effect of micronutrients on CRC as a primary outcome, however, CRC results were reported from secondary analyses. Only two of these trials identified nominally significant findings for CRC–one harmful, the other beneficial. The Alpha-Tocopherol Beta-Carotene (ATBC) Cancer Prevention Trial results for the intervention period were null for beta-carotene treatment [18, 42], however, the late post-trial follow-up period showed 88% more CRCs in the group randomized to beta-carotene [42]. The Nutritional Prevention of Cancer (NPC) trial tested selenium in the prevention of skin cancers and found a 58% reduction in incident CRC [43]. None of the other micronutrients tested in these 14 trials (ie, alpha-tocopherol, vitamin A, calcium, vitamin D, folate, B6, B12, vitamin C, or multivitamins) demonstrated an effect on CRC incidence.

The NIT treatment in the current analysis that reduced risk of CRC included the combination of selenium, alpha-tocopherol, and beta-carotene. As shown in **S7 and S8 Tables in S2 File**, a total of three different prior chemoprevention trials tested selenium, two to prevent colorectal adenomas (the Selenium and Celecoxib Trial, and the SELECT Ancillary Study) and two to prevent CRC (NPC and SELECT). The only effect on CRC (or its precursor) observed from these trials was the aforementioned reduction in CRC in the NPC [43]. This finding was based on preliminary results with limited follow-up and a total of only 27 CRC cases. A subsequent publication from this trial with an additional year of follow-up and one additional CRC case showed attenuation of the initial finding such that the P-value was no longer below the 0.05 nominal significance threshold. Results from the SELECT study with approximately five times more CRC cases than the NPC were null for selenium in both the active intervention [44] and post-trial follow-up periods [45].

For alpha-tocopherol, just one trial (the SELECT Ancillary Study) evaluated its effect on colorectal polyps, with null findings (**S7 Table in S2 File**). A total of six previous cancer trials tested the effect of alpha-tocopherol on the development of CRC (ATBC, WHS, HOPE/HOPE-TOO, WACS, SELECT, and PHS II), but all reported null results **(S8 Table in S2 File)**. Finally, beta-carotene was not included in any of the colorectal polyp prevention trials identified here, but four cancer prevention trials did report results on beta-carotene to prevent CRC (ATBC, PHS, CARET, and WACS). No effect of beta-carotene on CRC was seen beyond the increased number of CRC in the beta-carotene group described above in the late post-trial follow-up of the ATBC [42].

An interesting feature of the NIT results regarding the effect of the selenium/alpha-tocopherol/beta-carotene combination on CRC was that benefit was seen in females but not in males. For comparison, five of the micronutrient trials to prevent CRC (**S8 Table in S2 File**) enrolled both males and females, but just three described gender-specific results. Due to limited numbers of CRC in these trials, however, only total incident cancers and/or total cancer deaths were reported. The NPC reported a one-third reduction in total cancer incidence in males (P = 0.005) who received selenium but null results in females [46]. In contrast, the combined NORVIT/WENBIT observed a 27% increase in cancer incidence in male recipients of folic acid (vs no folic acid; 95% CI 1.07–1.51) with null results in females [47]. For VITAL, vitamin D showed no effect for all trial participants, males, or females for total cancer incidence [48].

The present study has a number of strengths as well as several limitations. This study is based on a large cohort of rural Chinese who participated in a 5.25-year randomized intervention trial that had an additional 25 years of post-trial follow-up. Compliance for pill taking throughout the intervention was excellent and follow-up during the 30-year intervention plus post-intervention follow-up periods was exceptional with very little loss to follow-up. Endpoint ascertainment was also considered complete as a result of the monthly contacts by the village health workers and the quarterly checking with the local cancer registry. Although the intervention was well-powered to test the primary hypothesis for the trial (reduction of esophageal/gastric cardia cancer incidence and mortality), the number of CRC cases diagnosed in this cohort was small compared to the much more common upper gastrointestinal cancers, thus, our power to evaluate intervention effects on CRC was limited. The people of Linxian were nearly all peasant farmers in the mid-1980s when this trial was initiated, and food quality and diversity were very restricted, resulting in limited frequency and variability in their diet. Our baseline study assessments (questionnaire and physical measurements) were rudimentary and did not capture some measurements that today are known to be important in the etiology of CRC (eg, physical activity, specific measures of body size). In addition, we had only a single baseline dietary assessment for our analysis of diet as a risk factor. Finally, the results from this unique, undernourished, rural Chinese population may have limited generalizability to the broader and increasingly urban Chinese population or to the Western world.

The present study is among the first RCTs to test micronutrients in the prevention of CRC in China, and is a unique evaluation of both risk factors and effects of micronutrient supplementation on the cause and prevention of CRC in a large cohort of rural Chinese over a three decade era that coincides with a period of extraordinary economic progress in China accompanied by the rapid rise of Western cancers including CRC. This initial characterization of the emerging CRC problem in China shows that risk factors for CRC in rural China share both similarities and differences with Western and urban Chinese populations. Perhaps most important, findings from the intervention suggest a preventive role for supplementation with the micronutrient combination of selenium/alpha-tocopherol/beta-carotene in the prevention of CRC, in this undernourished population, particularly in females.

## Supporting information

S1 Checklist (DOC)

S1 File (PDF)

S2 File (DOCX)
