# Supplementary material for: Colorectal cancer in the Linxian China Nutrition Intervention Trial: Risk factors and intervention results
Source: PLoS One. 2021 Sep 15;16(9):e0255322. doi: 10.1371/journal.pone.0255322 (PMC8443060; doi:10.1371/journal.pone.0255322)
Supplement: S1 File — (PDF) [file pone.0255322.s002.pdf]

PROPOSAL PROTOCOL

NUTRITION INTERVENTION STUDIES IN LINXIAN

19 March 1984

BETWEEN THE

CANCER INSTITUTE,

CHINESE ACADEMY OF MEDICAL SCIENCES

BEIJING, PEOPLE'S REPUBLIC OF CHINA

AND THE

NATIONAL CANCER INSTITUTE

BETHESDA, MARYLAND

Research Protocol for The Intervention Trial

Table of Contents

Page

A. Specific Aims 3

B. Significance of the Proposed Research 3

C. Pilot Intervention Study 5

D. Experimental Design 10

1. Participant Selection 10
2. Pre-Study Requirements 11
3. Intervention Assignment 11
4. Intervention Plan 12
5. Toxicity 13
6. Participant Compliance 16
7. Measures of Efficacy 17
8. Duration of Treatment 19
9. Statistical Considerations 19
10. Records To Be Kept 22

E. Human Subjects Protection Procedures 23

F. References 28

G. Appendices 32

A. Specific Aims

We propose to conduct two intervention trials using multiple vitamin-mineral supplements to evaluate the relation between such supplements and esophageal cancer mortality. One trial will be conducted in patients diagnosed with esophageal dysplasia and the other in the general population. We plan, in addition, to evaluate the effect of these supplements on regression/progression of dysplasia, esophageal and total cancer incidence, total cancer mortality, and total mortality.

To test the hypothesis that esophageal cancer mortality is related to nutrient intake, we will provide daily vitamin-mineral supplements to individuals and follow them for 5 years for the development of cancer in a randomized, doubled-blind, controlled intervention. These two studies will be conducted in Linxian (Henan Province) in the People's Republic of China (PRC). Linxian, a rural county with population 800,000, was selected because it has the highest rate of esophageal cancer in the world, and because there is suspicion that the population's chronic deficiencies of multiple nutrients may be etiologically involved.

Based on prevailing rates, over 1000 esophageal cancer deaths are expected in the populations to be studied. Over the 5-year study period, there will be greater than 90% power to detect a 40% reduction in esophageal cancer mortality among treated dysplasia patients, and similar power to detect a 20% reduction in the treatment group from the general population. The finding of substantially lower esophageal cancer mortality rates in the treated groups would provide strong evidence that minerals and/or vitamins are protective against cancer in humans.

B. Significance of the Proposed Research

The highest worldwide incidence and mortality for cancer of the esophagus occurs in the People's Republic of China. A nationwide survey of mortality conducted there in 1973-75 identified esophageal cancer as the second leading cause of cancer death, accounting for 27% of all cancer deaths in men and 20% in women.(1) The survey showed distinctive geographic variation within the country, with a 670-fold gradient between counties with the highest and lowest age-adjusted rates. Although elevated mortality is seen in several parts of the country, the most prominent cluster forms in north-central China, particularly in the Taiheng mountain area on the border of Henan, Hebei, and Shansi Provinces. Within this area in Henan Province is Linxian, a county with a population of 800,000 where the crude rates per 100,000 were 136 in males and 104 in females during 1977-82, and where the cumulative death rates to age 75 for esophageal cancer exceed 20% in both sexes.(2) Although there are only limited historical data, it appears as if esophageal cancer has long been a problem in these areas and that there has been little change in the death rates from 1959-82.

Abnormalities thought to represent esophageal cancer precursor lesions are common in Linxian. Past cytologic and endoscopic surveys in Linxian indicate that only about 20% of the adult population have "normal" esophageal epithelial. (3) The remaining 80% have some degree of esophagitis, including approximately 20% with esophageal dysplasia.

Previous Studies of Esophageal Cancer in China

Numerous epidemiologic and laboratory investigations have been conducted in areas of China at high and low risk of esophageal cancer.(3-10) We have twice organized multidisciplinary surveys to assess levels of environmental factors (e.g., trace elements in water, food, sera, urine, and hair; nitrates in well water; consumption of pickled vegetables, moldy breads, and other foods; nutrient levels in blood) for brigades, communes, or counties in relation to the esophageal cancer rates for these geographic units.(4-6) Case-control interview studies have been conducted in Xinjiang and Jiangsu Provinces.(4) The most intense analytical study, however, has been in Linxian, where the characteristics of esophageal cancer and its precursor lesions have been under study for the past 25 years.

The studies have provided a series of clues to etiology, but have not yet conclusively pinpointed a specific carcinogen responsible for the elevated esophageal cancer rates. They have identified significant differences, noting that the high-risk areas tend to be rural and poor, with dry climates, infertile soils, and water often in short supply.(4) Diets have been typically low in fruits and vegetables. There appear to be widespread deficiencies of multiple nutrients (riboflavin in particular, but also vitamins A and C, zinc, molybdenum and others).(3,7) The previous studies suggest that specific foods commonly eaten in Linxian and other areas may contribute to the initiation of the tumors. Under suspicion are pickled vegetables and other foods that also become fermented or moldy; including moldy breads and cheeses in Xinjiang.(4-6) Silica fragments, most likely from ingested millet, have been found in esophageal tissue from cancer patients.(8) Nitrosamines have been detected in several of these foods in high-risk areas, and Ames tests have revealed evidence of mutagenicity in samples of the pickled vegetables.(4,9,10) One small experimental study conducted in China also showed that cancers of the esophagus and forestomach developed in rats fed moldy cornbread, while another showed that fungal contamination could promote the synthesis of nitrosamines.(9) Esophageal fungal infections were diagnosed in the majority of patients with esophageal cancer in one survey in Linxian.(4) The previous studies indicate that smoking and drinking -- the major determinants of esophageal cancer in Western societies -- may play some role in China, but relatively minor ones. Familial clustering has also been observed, but it seems likely that the high cancer rates are predominantly of environmental origin.

We are currently conducting a case-control study on esophageal cancer in Linxian in collaboration with NCI. This study (supported by contract number N-CP2-1012) will

more fully evaluate the etiologic role of multiple risk factors, such as moldy or fermented foods, smoking and drinking, family history of cancer, exposure to nitrosamines, and other factors. The case-control study will also obtain dietary histories and attempt to assess micronutrient status in cases compared to controls. However, the role of micronutrients status can best be evaluated via a prospective experimental trial. Thus, we are pleased to respond to NCI's request to conduct an intervention trial to test the hypothesis that vitamin/mineral deficiency is related to the high risk of esophageal cancer in Linxian.

As noted, the reasons for the exceptional risk of esophageal cancer in certain areas of north China are not yet known, but they may be related to increased exposure to specific carcinogens among a population unusually susceptible because of chronic nutritional deficiencies. Consistent evidence is now emerging from a number of epidemiologic investigations conducted outside China that patients with epithelial cancers, including those of the esophagus, often had diets characterized by reduced intakes of certain foods, particularly fruits and vegetables. Epidemiologic studies conducted in the United States(11-13), Puerto Rico(14), Japan(15), Iran(16), and South Africa(17), all have shown relatively strong associations between esophageal cancer risk and poor nutrition. Laboratory evidence suggests that specific nutrient deficiencies may be responsible for these associations. Low levels of vitamin A are suspect since retinoids have marked anti-promotion properties in experimental studies.(18) An independent influence of pro-vitamin A (beta-carotene) has also been postulated on biologic grounds.(19) Riboflavin deficiency can produce esophageal epithelial changes and affect carcinogen metabolism, and enhance carcinogenesis (20-21), while vitamin C is thought to block the formation of nitrosamines (22), and vitamin E to act as an antioxidant and inhibitor of chemical carcinogenesis (23). Mineral deficiencies also can modify cancer incidence. Zinc deficiency has been shown to enhance methylbenzyl-nitrosamine-induced esophageal carcinogenesis in rats.(24) Zinc also influences the release of vitamin A from liver stored in humans, with serum concentrations of retinol tending to be low in persons with low levels.(25) Studies in our laboratories have shown that molybdenum can inhibit cancers of the forestomach in rats.(26)

In summary, the strong weight of evidence from these experiments suggests that several vitamins and minerals can have a pronounced effect on tumor development. Combined with the results of the epidemiologic investigations, an hypothesis emerges that low intake (although not necessarily low enough to induce frank clinical deficiencies) of nutrients can enhance risk. Thus vitamin and mineral supplementation among persons with low levels of consumption of several nutrients and with high rates of cancer may result in a reduction in cancer risk.

C. Pilot Intervention Study

The CICAMS has been concerned with the problem of esophageal cancer in Linxian since 1959, when a field station was established there to improve diagnosis, treatment, and public health work aimed at the prevention of esophageal cancer. In conjunction with the Linxian Esophageal Cancer Research Institute and the Henan

Province Cancer Institute, the CICAMS has maintained for several years an active, reliable surveillance system and registry of esophageal cancer in Linxian. Our long commitment to Linxian has earned the trust and cooperation of local institutions.

The nutritional intervention trials proposed herein will build directly on the experience of a pilot study begun in October 1982, under an NCI contract (N-CP2-1012) between the CI, CAMS, and the U.S. NCI.

Pilot studies were undertaken to assess the feasibility of large scale interventions. The specific questions the pilot studies were designed to answer included:

(1) Can sufficient numbers of persons form the general population be recruited?

(2) Can sufficient numbers of dysplasia patients be identified and recruited?

(3) How compliant will participants be?

(4) Can the adminstrative logistics of patient recruitment, information collection, pill delivery, and follow-up be managed?

(5) What is the current nutritional status of the population?

(6) Will supplementation with U.S. Recommended Daily Allowance (RDA) levels of vitamins improve low or deficient states?

Two pilot trials were conducted, the first involving the general population and the second involving persons diagnosed with severe esophageal dysplasia.

For the general population pilot, 21 production teams (villages) were randomly selected from two communes (Yaocun and Rencun) in northern Linxian. A central census roster listed 937 names of persons aged 40-69. Sixty-two (7%) proved to be ineligible for the following reasons:

- suffering from esophageal cancer - 7

- suffering from other cancers - 2
- suffering from other debilitating disease - 5
- had already died - 18
- temporarily assigned work outside the county - 16
- other (incorrect age, erroneous or duplicate listing, etc.) - 14

852 of the 875 eligible subjects (97%) agreed to enroll in the general population pilot trial. For the dysplasia pilot trial, 91 persons from Yaocun and Rencun communes most recently discovered by balloon-swallow cytology examination to have severe dysplasia were recruited. All 91 agreed to participate. Criteria for the diagnosis of dysplasia are given in Appendix 1. All participants in the pilot studies were given One-A-Day (Miles Laboratory) multiple vitamin tablets for 6 months.

Barefoot doctors visited the subjects during the trial to distribute pill packs. Table 1 shows the losses to follow-up during the course of the trial. Losses were generally due to death, cancer, or other debilitating diseases occurring among participants, or to movement away from the study area. At the end of the 6-month trial, losses were about 3%.

Table 2 gives the percent of enrolled participants who refused to take any of the distributed pills. At the end of the study period, these refusal rates were 2.5%. Among those taking at least some pills, over 95% (as judged by counting unused pills) were taking the large majority of the pills by the end of the trial (Table 3). In addition to counting unused pills, compliance were administered loading-dose tests for urinary riboflavin prior to pill-taking and at either 3, 5, or 9 weeks after the start of supplementation. Analysis of the previous data is still ongoing, but Table 4 shows that average levels of riboflavin were markedly higher after than before supplementation.

Table 1. Cumulative Percent Lost to Follow-up
During the 6-Month Pilot Intervention Trial

Month	Percent
1	1.6
2	1.7
3	1.7
4	3.1
5	3.1
6	3.3

Table 2. Cumulative Percent of Participants Refusing
to Take any Pills During the 6-Month Pilot Intervention Trial

Month	Percent
1	0.0
2	2.4
3	2.4
4	2.4
5	2.5
6	2.5

Table 3. Monthly Percent of Participants Taking at Least 80% of the 6-Month Pilot Intervention Trial

Month	Percent
1	92.3
2	88.6
3	93.9
4	97.6
5	96.8
6	96.1

Table 4. Mean Riboflavin in 4-Hour Urine Samples Before and During the Pilot Intervention Trial

Time of Specimen Collection	n	x (mg)
Before	116	756
After 3 weeks	40	1362
After 5 weeks	44	1423
After 9 weeks	32	1368

Nutritional assessments involving laboratory assays of blood and urine were conducted prior to the start of and 4 months after supplementation. The assessments were carried out on 100 randomly selected individuals taking daily pills and (as a control) on 51 similarly-aged individuals from commune production teams not participating in the pilot study. High-pressure liquid chromatography (HPLC) analyses of serum for retinol, beta-carotene, and tocopherol have been conducted, and red blood cells have been assayed for glutathione reductase activity (as a more sensitive measure of riboflavin status than provided by the urine tests). Plasma and urinary ascorbic acid and urinary niacin and thiamine are also being determined. Results are not yet fully available, but preliminary data from the HPLC analysis (conducted at the New Jersey

Medical School in the laboratory of Professor C. S. Yang) show sizeable shifts toward increasing status for retinol and vitamin E (both included in the One-A-Day pills), but not beta-carotene (not included in the pills).

The pilot study thus suggests that a vitamin intervention trial will be well accepted in Linxian. Indeed compliance, as judged by the pill counts, was exceptionally high. Of course, the disappearance of a pill from the pill pack does not guarantee that the pill was actually taken by the participant, but our initial laboratory analyses provide biochemical confirmation from both urine tests (reflecting recent pill taking) and blood tests (reflecting longer-term pill taking) that compliance was high. The pilot study also led to the establishment of an organizational system which can be readily expanded to accommodate a larger trial.

In summary, progress to date indicates that a population-based nutritional intervention study of esophageal cancer in Linxian is feasible. Pilot Study results have clearly shown that: patients can be identified and recruited; compliance is excellent; administrative logistics can be handled; nutritional deficiencies are numerous; and low dose vitamin supplementation improves nutritional status.

D. Experimental Design - Intervention Plan

1. Participant Selection

Inclusion Criteria (both interventions):

a. Limited to 3 northern communes (Yaocun, Rencun, and Donggang).

b. Males and females aged 40-69.

c. Able and agree to participate for duration of trial.

In addition, for the dysplasia trial only, persons must have been found to have severe (group 1 or 2) dysplasia in a mass balloon cytology screening conducted in the fall of 1983.

Exclusion Criteria.

a. Currently taking vitamins regularly.

b. Malignancy or other debilitating disease.

c. Taking certain specific medications (e.g. the traditional drug, Anti-Tumor B3 or synthetic retinoids).

2. Pre-Study Requirements

a. Complete a pre-study evaluation which includes having a physical examination and filling out a short questionnaire asking about demographic information and esophageal cancer risk factors (see Appendix 2).

b. Give informed consent.

3. Intervention Assignment

Dysplasia Component

Participants will be recruited from among residents of Yaocun, Rencun and Donggang communes identified as having severe esophageal dysplasia in the December 1983, balloon- swallow cytology screening. The identification and interviewing of dysplasia patients is expected to take one month. All eligible dysplasia subjects who complete the pre-study requirements will be randomized by individual in blocks of 8 into treatment (or placebo) groups, stratified by age (5-year groups), sex, and commune. Actual enrollment of subjects for the dysplasia trial is expected to take an additional 2 months to complete.

General Population Component

Participants will be recruited from census lists of eligible residents aged 40-69 from three communes (Yaocun, Rencun, and Donggang) in northern Linxian (excluding those already taking part in the dysplasia component). Census figures indicate that the current population of the 3 target communes for males and females age 40-69 is 33,643.

The pre-study evaluation for these subjects is expected to take 2-3 months and will be started after the dysplasia trial accrual is complete. Those eligible and completing pre-study requirements will be randomized by individual in blocks of 8 into treatment (or placebo) groups, stratified by age (5 year groups), sex, and commune. Accrual for the general population trial is expected to take 4 months to complete.

For both the dysplasia and the general population components, randomization will be conducted in Beijing and the key will be kept at NCI so as to keep the trial double blind for all involved persons in Linxian and Beijing.

4. Intervention Plan

The type and dosage of vitamins and minerals to be administered will differ for the two trials. For both trials, participants will receive various combinations of vitamin A, beta-carotene, riboflavin, niacin, vitamin C, vitamin E, selenium, zinc, and molybdenum, in doses between one and three times the U.S. Recommended Daily Allowances (RDA). The RDA for the nutrients with National Academy of Sciences-specified-levels are:(27)

vitamin A - 5000 I.U., riboflavin - 1.6 mg, niacin - 18 mg,

vitamin C - 60 mg, vitamin E - 10 mg, and zinc - 15 mg.

Different study designs will be used for the two trials. The dysplasia trial will use a simple two-group design with one-half the patients assigned to receive multivitamin-multimineral pills to be taken daily. The other one-half participants will receive placebos identical in appearance to the active pills (see Table 5 for exact dosages).

For the general population trial, a factorial design will be used. The form of design to be used is a one-half replicate of a 2X4 factorial design.

The 4 proposed factors include:

A - vitamin A, beta-carotene, zinc

B - riboflavin, niacin

C - vitamin C, molybdenum

D - selenium, vitamin E

A full 4-factor factorial design would include 2X4 = 16 different treatment groups (shown below).

<u>Placebo</u>	A	B	<u>AB</u>
C	<u>AC</u>	<u>BC</u>	ABC
D	<u>AD</u>	<u>BD</u>	ABD
<u>CD</u>	ACD	BCD	<u>ABCD</u>

One-half repetition includes only 8 treatment groups (the 8 underlined above).

This design was chosen because it will allow us to separate the main effects of various grouped factors (and certain of the interactions between factors) without requiring the excessive number of treatment groups needed for a 24 full factorial design or a factorial design evaluating greater numbers of factors. Further discussion of the rationale behind this choice of design is given in Appendix 3.

All treatment groups (including placebo) will have a small amount of thiamine (0.375 mg or 25% RDA) included. The addition of thiamin allows us to provide a small amount of a vitamin potentially useful for general health status, but which by itself would be unlikely to influence esophageal cancer incidence or mortality.

It is anticipated that the pills for both trials will be formulated in the United States and packaged in pill packs in China.

The barefoot doctors will deliver the vitamin pills in packs containing a 4-week (28-day) supply. At the end of each 28-day period, the barefoot doctor will collect the used pill pack and give out a new pack. Each will be labelled with the subjects study number and name.

Before starting the trials, a manual of field operations for interviewers, pill distributors, and other field staff will be developed and training sessions will be held to assure that standardized procedures are adhered to in all study procedures, including interviewing, distribution of pills, monitoring of compliance, and documentation activities.

5. <u>Toxicity</u>

No toxicity is expected in administration of vitamins and minerals given at 1-3 times RDA levels. The proposed levels of micronutrients are shown in Tables 5 and 6 below

Table 5. Proposed Dosages, Dysplasia Trial

Nutrient	Dose	%RDA
Vitamin A (as acetate)	10,000 IU	200
Beta-carotene	25 mg	-
Riboflavin	5.2 mg	300
Niacin (as niacinamide)	40 mg	200
Vitamin C (as ascorbic acid)	180 mg	300
Vitamin E (as dl-alpha tocopherylacetate)	20 mg	200
Selenium (as sodium selenate)	50 mcg	-
Zinc (as zinc sulfate)	45 mg	300
Molybdenum (as sodium molybdate)	30 mcg	-

Table 6. Proposed Dosages, General Population Trial

Nutrient	Dose	%RDA
Vitamin A (as acetate)	5000 IU	100
Beta-carotene	25 mg	-
Riboflavin	3.2 mg	200
Niacin (as niacinamide)	40 mg	200
Vitamin C (as ascorbic acid)	120 mg	200
Vitamin E (as dl-alpha tocopherylacetate)	20 mg	200
Selenium (as sodium selenate)	50 mcg	-
Zinc (as zinc sulfate)	22.5 mg	150
Molybdenum (as sodium molybdate)	30 mcg	-

These doses are well within the level of intake in the American population as a whole, for which no long-term toxicity has been reported.

The water-soluble vitamins are rapidly excreted with essentially no short-or long-term toxicity reported.

The fat-soluble vitamins are stored in the body for a longer time and have the potential for toxicity. However, no vitamin A toxicity has been reported for long-term intakes of less than 50,000 IU or 15,000 retinol equivalents [1 R.E. of retinol = 3.33 I.U. and 1 R.E. of Carotene = 6 mcg.] daily.(28) Beta-carotene has no known serious side effects although, depending on body size and dietary carotene intake, it may cause reversible yellowing of the skin. With the possible exception of muscle pain, vitamin E has no documented toxicity, even in doses in excess of 400 IU/day. (29)

Selenium has been reported to cause toxicity in humans but only when daily intake exceeds 3 mg (60 times the dose to be given).(30)

Acute zinc toxicity can occur at doses around 2 gm but doses of 150 mg daily for years have not produced toxicity.(31)

Molybdenum has not been reported to cause long-term toxicity in doses below 0.50 mg/day.(32)

Though unexpected, allergic reactions to fillers or coatings of vitamin pills could occur.

Barefoot doctors will be instructed in the signs and symptoms of potential adverse reactions to the pills. While no specific check-list will be systematically reviewed with participants at each monthly visit, patients will be asked to report any change in their general health status. If an allergic reaction or other potential drug effect is suspected, the pills will be stopped. Patients may be re-challenged in one month if the reaction was mild and reversible.

No provision is made for dose modification. Patients with persistent or recurrent toxicity suspected of being drug-related will not be given additional pills but will be followed for the duration of the trial for the outcomes of interest.

6. Participant Compliance

For both the dysplasia and general population groups, compliance will be assessed in two ways: pill counts for all participants, and biochemical tests for a sample of them.

The barefoot doctors will distribute the monthly (28-day) pill packs. Each pill pack will be labelled with the participant's study ID number and name. Barefoot doctors will visit participants weekly for the first month of the study, then monthly thereafter. At the end of each month (28-day period), the barefoot doctor will collect the used pill pack and give out a new pack. A sample of the retrieved pill packs will be analyzed to assure us that the participant received the pills he was assigned to receive. Each month he will record in a log book the number of pills remaining for each subject. Logs of all contacts with the persons enrolled in the trial will also be maintained by the barefoot doctors. Bi-monthly sessions with participating barefoot doctors will be held to compile compliance statistics and to identify any practical problems that may arise.

Compliance will also be monitored using biochemical tests in a sub-sample of the participants. Approximately 400 randomly selected study subjects per year will be checked for compliance in each of the 5 years. Groups of 100 will be assessed at quarterly intervals. Each group of 100 will be composed of 20 dysplasia and 80 general population subjects. Compliance testing will be conducted as a part of the biochemical surveillance program (see 7 - Measures of Efficacy). In addition to measurements outlined there, erythrocyte glutathione reductase activation coefficients will be measured as an indicator of riboflavin status. Measuring riboflavin status is particularly appropriate as a check on compliance since dietary intake of riboflavin is depressed in over 90% of the Linxian population. Thus an adequate level is a good indicator that participants are taking pills. Compliance checks will be conducted without forewarn-

ing and records will be managed in such a way that results of these compliance checks may not be used to unblind the treatment group assignment for any subjects or study personnel.

7. Measures of Efficacy

Esophageal cancer mortality over a 4 1/2-year period beginning 6 months after the start of the nutritional supplementation will be the primary outcome variable to be measured in the dysplasia trial and the population trial. Total mortality, total cancer mortality, and esophageal and total cancer incidence will also be assessed. Several ancillary assessments will also be made. These include: 1) reviewing cytologic regression and/or progression in all patients in the dysplasia trial, 2) doing DNA content analysis on a sample of these cytologic slides, 3) endoscoping a sample of 200 dysplasia patients before starting pills and again after 18 months of pill-taking to assess histologic changes and correlate the histology and cytology findings, and 4) obtaining 24-hour urine collections before and after the start of pill-taking to evaluate the effect of vitamin C on nitrosamine formation. (See Appendix 3 for more details on these ancillary assessments).

During the course of the study barefoot doctors will identify all deaths to study subjects. Information from medical records will be abstracted to include date and cause of each death. Incident cancer cases will also be identified. Date of diagnosis and type of cancer will be determined. Annually, in collaboration with NCI, the CI will calculate age- and sex-specific rates of mortality from all causes and rates of mortality and incidence from esophageal and all other cancers among study participants, stratified by treatment group (with the code identifying placebo and treatment groups broken only if there are highly significant differences).

A biochemical surveillance program will be included in both trials to provide information on background of nutrients for trial participants before the interventions, to assess compliance, and to allow us to measure seasonal and secular variation during the interventions. To accomplish this, all patients participating in the dysplasia trial and a sample (approximately 10%) from the general population trial will be asked to provide samples of blood, urine, hair, and nails prior to the onset of pill-taking.

During the interventions, 400 study subjects per year will be selected at random to have blood and urine collected and have a dietary history and anthropometric measurements taken. Blood will be analyzed for the various vitamins and minerals being administered during the trials and urine will be analyzed for nitrosamines (see Table 7 below). This surveillance will include participants from both trials limited to those who had samples collected before the onset of pill-taking and will be performed throughout the year (100 persons every 3 months; 20 from dysplasia trial, 80 from general population trial).

The laboratory analyses for this surveillance program will be performed in China with a 10% sample being sent to the U.S. for simultaneous analysis as a part of an ongoing quality control program.

In addition to assessing compliance and seasonal/secular changes, baseline measurement of nutrients in all dysplasia trial participants will allow us to adjust for any non-random differences between treatment groups and review our outcomes by baseline nutrient status.

Table 7

Laboratory analyses to be conducted during the trials.

Analyte	Tissue	Method
Retinol	Plasma	HPLC
Carotenoids	Plasma	HPLC
Tocopherol	Plasma	HPLC
Riboflavin	Erythrocytes	Glutathione reductase activity
Niacin	Urine	Spectrophotometric
Ascorbic acid	Plasma	Spectrophotometric
Molybdenum	Erythrocyte, hair, nails	Spectrophotometric
Selenium	Erythrocyte, hair, nails	Spectrophotometric, neutron activation
Zinc	Plasma, hair	Spectrophotometric
Cholesterol	Plasma	Spectrophotometric

8. Duration of Treatment

The anticipated duration of treatment will be 5 years for both the dysplasia component and the general population component. Relying on existing population rosters and on the Linxian esophageal cancer registry, it will be possible to follow total mortality and total esophageal cancer incidence and mortality for the entirety of both cohorts, including those who never enroll and those who drop out of the study.

9. Statistical Considerations

(a.) Sample size

The total population aged 40-69 in the three communes in which the study will be conducted numbers 33,643, based on the national census conducted in China in 1982. In December 1983, a mass screening of esophageal cytology by balloon swallow was conducted by the CI among 12,000 adults age 40-69 in these communes. The cytology slides are still being scored, but preliminary results indicate that 20% of those screened have severe esophageal dysplasia (see Appendix 1, Cytologic Diagnosis of Dysplasia). At this percentage, approximately 2,400 dysplasia patients will be identified as potential subjects for the dysplasia trial.

It is proposed to enroll all eligible persons in the general population and in the dysplasia trials. From our pilot study, we estimate that 90% will be eligible and willing to participate in the trials. Thus, there will be 2,160 participants in the dysplasia trial and approximately 28,000 [(33,643-2,400) x .9] in the general population trial.

(b.) Power

The following assumptions were made in the power calculations:

(1) The annual crude esophageal cancer mortality rate in persons aged 40-69 in the general population trial is 650 x 10-5 (or 0.65% per year);

(2) The annual mortality from esophageal cancer in patients with severe dysplasia age 40-69 is 1.78%;

(3) Excluding events in the first 6 months, the rates in the following 4 1/2 years will be 2.9% for the general population and 8.0% for those with dysplasia.

(4) The effect of drop-outs and competing risks is ignored; and

(5) Alpha error (one-sided) is set at 0.05.

For the dysplasia trial, with Po = .08 and 1,080 persons in each treatment arm; the detectable differences and power are shown below:

1.6%/yr

P1	Decrease due to intervention	Power
0.064	20%	42%
0.060	25%	57%
0.056	30%	72%
0.052	35%	84%
→ 0.048	40%	93%
0.044	45%	97%

For the general population trial, with Po = .029 and 14,000 per treatment arm and assuming a 2 group trial for simplicity of calculation; detectable differences and power are:

P1	Decrease due to intervention	Power
0.0247	15%	72%
→ 0.0232	20%	92%
0.0218	25%	99%

These estimates are based on the formula (see e.g. ref [33]).

(INSERT FORMULA)

$$\phi = \left[\frac{-Z_{\alpha}(A+B) + \sqrt{N_0 (A+B)} \, |P_1 - P_0|}{\sqrt{(A+B)(RA-B) - R(P_1-P_0)^2}} \right]$$

whereP1 = outcome proportion in the index series

Po = outcome proportion in the comparison series.

R = ratio of size of comparison series to size of index series

No = size of comparison series

Za = value of standard normal deviate corresponding to desired alpha-level of the test

A = P1(1-Po) = Po(1-P1)

B = (R-1) Po(1-Po)

ϕ = distribution of a standard normal deviate(32)

Thus, the dysplasia trial will be able to detect with high power a 40% reduction in esophageal cancer rates associated with the intervention, and the general population trial, a 20% reduction. The power of the general population trial (which will use a fractional factorial design---see Section D.4) to detect differences associated with individual factions will vary under various possible alternative outcomes, but will be high to evaluate differences of 20-30% due to each factor under a number of plausible outcomes. Because the number of deaths due to esophageal cancer will be lower than the number of incident esophageal cancer cases, total cancers, and total mortality, power for looking at these other outcomes of interest (not shown here) will be greater.

(c.) Data Analysis

The methods of analysis to be applied to the data collected during these trials include simple contingency table analysis and Cox's method of regression analysis for survival data.(34) The use of regression methods will allow for incorporation of variables stratified on at randomization and adjustment for risk factors which are found by the initial interview to be nonrandomly distributed between treatment groups.

Analysis of the dysplasia trial data will allow us to make a statement about the effect of the combined multivitamin-multimineral preparation only, while in the general population trial we will be able to separate out the effects of the several factors.

(d.) Stopping Rule

The advantages of a single final analysis of the data and sequential analysis will be combined in a policy of examining the data annually, but not stopping prematurely unless the difference between treatments reaches some extreme level of statistical significance

(e.g., p.001).(34) Otherwise, we will let the trial run its full course of 5 years at the end of which ordinary statistical analysis will be undertaken.

10. Records to be Kept

The CICAMS will develop, in collaboration with NCI, questionnaires and forms needed for the collection, storage, and coding of data. All questionnnaires and recording forms used in the study will be developed in both Chinese and English-language versions, and will be reviewed by NCI before being used in the study.

Questionnaires and forms will include the following:

1) a brief questionnaire for all study participants, containing personal identifiers, demographic information (age, sex, production brigade), information necessary to confirm the subject's eligibility (for example, whether the person is taking vitamin supplements or other medicines regularly), and several items concerning risk factors such as family history of esophageal cancer patterns of consumption of pickled vegetables or moldy foods and cigarette smoking status;

2) a cytology abstract and follow-up form for all those included in the 1983 balloon-swallow mass screening, with space for such information as name, date of birth, sex, commune of residence, study ID if eligible for study, initial cytological diagnosis, and any subsequent cytological diagnosis during the course of the study;

3) a form to collate information on all persons considered for the trials, with space for such information as name, date of birth, sex, production brigade of residence, study ID if eligible (or reason ineligible), whether enrolled (or reason if not enrolled), biochemical test status and results, compliance information during the course of the study, and vital status at the completion of the study, with date and cause of death if deceased;

4) logs necessary to perform such tasks as:

assignment of identification numbers, randomization, enrollment, tracking, and distribution of pill packs, reclamation of used pill packs, chronicling of barefoot doctors' visits with participants, noting of pill counts for the purpose of compliance assessment, and recording of dates and causes of death;

5) questionnaires and recording forms needed:

a) to conduct a biochemical surveillance program to monitor secular and seasonal changes in nutritional status and diet within and among treatment groups, and b) to perform biochemical tests for the purpose of compliance assessment;

6) coding sheets listing all of the data collected for each individual, reviewed and edited for completeness and accuracy;

7) logs necessary to maintain quality control of data, including:

a) maintaining a written log or record book of all decisions that affect the design, conduct, or analysis of the study;

b) monitoring the performance of all field staff including

1) repeat interviews by a supervisor for portions of each baseline questionnaire for 2% of all study subjects interviewed, and re-interview of all subjects interviewed by the original interviewer if major discrepancies are found; and

2) having the supervisor independently contact 2% of the participants to verify that the barefoot doctors have delivered the proper pill packs on schedule and have counted unused pills correctly;

c) verifying that all coding of data is accurate;

d) ensuring that all blood, urine, hair, and nail specimens are collected, processed, and stored in a manner that will enable valid analysis for nutrients;

e) ensuring that proper quality control procedures are carried out for all laboratory work, such as performing analyses in duplicate, constructing multiple-point standard curves, and -- simultaneously with the samples form study subjects -- analyzing internal standards, external standards, and "doped" standards to determine recovery, precision, and accurary;

f) recovering used pill packs with unconsumed pills left in the package. A random sample of these will be returned to the United States for chemical analysis, as a way of verifying the appropriate formulation and assuring that subjects are receiving the correct pills.

E. Human Subjects Protection Procedures

Procedures for the protection of humans from unnecessary research risks have been developed by the Chinese Academy of Medical Sciences. These are generally similar to NIH procedures described in the Belmont Report that are used in the United States. We will abide by the rules listed in the Belmont Report, and have the equivalent of an Institutional Review Board to monitor our procedures for subject protection.

These intervention trials involve minimal and acceptable risk. There is only minimal risk from having blood samples drawn, and our experience with the balloon cytology examination in over 35,000 individuals indicates that this procedure does involve increased human risk or any health problems. Endoscopy is a medically accepted procedure in Linxian, as elsewhere in China, with minimum and acceptable risk. Participa-

tion in the trials is not contingent on providing blood or other samples or on undergoing additional examinations once the trial has started. All participants in the trial will be voluntary participants. Our pilot study indicates that villagers in Linxian are eager to participate, and the great majority readily give informed consent. Even though they know that this is an experimental trial with no guarantee of success, they seem willing to join in the hopes that the study may provide new information about the causes of a cancer that they all know too well. Although written consent is not obtained in China for such studies (many older individuals brought up prior to liberation in Linxian cannot read or write), we will obtain consent after reading them the consent forms listed below.

Dysplasia Trial - Informed Consent

We are conducting a trial to see if we can reduce the number of people dying from cancer of the esophagus by giving them vitamins and minerals. If you choose to participate in this trial, you will first be requested to complete a questionnaire asking for certain information about your background, diet, and history of cancer in your family. Then you will be asked for a blood sample (10 cc), a urine sample (24 hour collection), a hair sample (small lock from back of head), and toenail clippings (from one big toe and one smaller toe). You may also be asked to take another examination, called an endoscopy, to further characterize your dysplasia. You do not have to provide any information or samples or undergo any additional exams to participate in the trial, but this information will be very useful to us in understanding more about how to prevent esophageal cancer.

If you participate, you will be given pills to take daily for 5 years. All the pills will contain vitamins or minerals, but in different amounts. Side effects from these pills are not expected but could occur. A barefoot doctor will visit you every month to give you a new supply of pills and see how you are. At the end of 1 1/2 years, 3 years, and 5 years, you will be asked to swallow the balloon again, and may be asked to have an endoscopic examination to see how the lining of your esophagus is doing. You may also be asked for another blood and urine sample after you have been taking your pills for a while.

Your participation in this trial is voluntary. If you start the trial, you are free to stop being a participant at anytime if you choose.

General Population - Informed Consent

We are conducting a trial to see if we can reduce the number of people dying from cancer of the esophagus by giving them vitamins and minerals.

If you choose to participate you will be asked for information on your background, dietary habits, and history of cancer in your family. You may then be asked for a sample of blood (10 cc), a urine sample (24 hour), a hair sample (small lock from back of head), and toenail clippings (from one big toe and one smaller toe). You do not have to provide any information or samples to participate in the trial but this information will be very useful to us in understanding more about how to prevent esophageal cancer.

If you participate you will be given pills to take daily for 5 years. All the pills will contain vitamins or minerals but in different combinations or amounts. Side effects from these pills are not expected but could occur. A barefoot doctor will visit you every month to give you a new supply of pills and see how you are doing. You may be asked for a blood and urine sample after you have been taking your pills for a while.

Your participation in this trial is voluntary.

E. Personnel

The CI will provide qualified scientific and support personnel to conduct the intervention trials in collaboration with NCI.

Key Personnel:

Dr. Li Bing, M.D., Vice Director, CI, who will be the Principal Investigator responsible for the direction of the trials. Dr. Li has been the director of research at the CI since 1958 and is responsible for the overall direction of all cancer research in the People's Republic of China. She has been involved in research in Linxian for over 20 years.

Dr. Li Jun Yao, M.D., Chief, Department of Epidemiology, CI, who will be the Associate Project Director. Dr. Li will assume daily responsibility for the conduct of the trials. He has over 15 years experience in management of epidemiologic studies in China, including the lead role in the nationwide survey of cancer mortality during 1973-75 in which the CI recruited approximately 600,000 field workers to ascertain all deaths due to cancer. He also has directed case-control studies of esophageal cancer in Xinjiag and Jiangsu provinces, and is the Associate Project Director for the ongoing case-control study in Linxian.

The CVs for Dr. Li Bing and Dr. Li Jun Yao are given at the end of this section.

Support Personnel:

In addition to the 2 key personnel, the CI will provide many clinical, field, and laboratory personnel to carry out these trials. The following individuals will be assigned to this project:

Dr. Li Guang Yi;Biochemist/epidemiologist;Field supervisor, responsible for day-to-day operations in Linxian

Dr. Liu Run-Yuan; Statistician ;Data manager, responsible for data preparation and transcription

Dr. Yu YuEpidemiologist; Co-Field supervisor, responsible for laboratory analysis of vitamins

Dr. Zheng Shu-Feng;Nutritionist;Co-investigator, responsible for laboratory analysis of vitamins

Dr. Wang Guo-Qing;Surgeon;Co-investigator,responsible for endoscopy to assess histologic changes

Dr. Lu Shi-Xin;Etiologist;Co-investigator,responsible for nitrosamine analysis of urine to evaluate the effect of vitamin C on nitrosamine formation

Dr. Yang Kan;Pathologist;Co-investigator,responsible for pathological diagnosis of esophageal cancer

We will also have 6 local supervisors (2 for each commune) stationed in Linxian at the start-up of the trials. These individuals will conduct training sessions with the barefoot doctors, supervise their activities during the pre-study interview of the dysplasia patients and all 33,000 members of the general population, and manage the collection, preparation and storage of blood and urine specimens. The CI will also send 3

specimen collectors and 4 lab technicians to assist in and ensure quality control of the biologic specimen collection. Two of the local supervisors, 1 specimen collector, 2 of the lab technicians, and 3 data abstractors will remain in Linxian during the course of the trial.

Barefoot Doctors:

The interviewing of participants for enrollment in the trials, the distributing of new pill packs, the collecting of used pill packs, and counting of unused pills, and the tracking of the participants for mortality will be done by barefoot doctors. The barefoot doctors will also assist in the specimen collection. Approximately 175 barefoot doctors will be recruited. The pilot study indicated that barefoot doctors could perform the tasts requested of them quite well, and that the organizational system involving supervision from CI field supervisors was effective. This system also incorporated medical staff from the 3 communes and the one Linxian County hospital, plus the Linxian Cancer Institute, so that the entire medical community provided support for the trial.

Consultants:

Several individuals or groups of consultants will be used to advise and assist in the execution of these trials. Although we have some experience in each of the laboratory tests mentioned in the protocol, our expertise in conducting some of the tests for the biochemical surveillance is not fully developed. We thus have asked Dr. C.S. Yang, Professor of Biochemistry at the New Jersey Medical School to serve as a consultant (Dr. Yang's C.V. is attached). Drs. Yang Shu-Ping and Sun of the CI have just finished a 5-month stay in the lab of Dr. C.S. Yang, where they gained much experience in the analysis of serum retinol, beta-carotene, and tocopherol, using high-pressure liquid chromatography (HPLC). Dr. Yang, who is an expert in nitro-samine as well as nutrient analysis will continue to assist during the course of the trials, including the establishment of a newer HPLC machine to replace the older, less sensitive version now at the CI. In addition, Dr. Yang will conduct analyses in the U.S. on 10% of the samples in the biochemical surveillance program in duplicate with us for purposes of quality control.

We will also rely on Dr. Shen Qiong as a consultant for the report on cytologic examinations to be given to persons enrolled in the dysplasia trial. Dr. Shen has pioneered the development of the balloon swallow cytologic technique. He will ensure that criteria used for the cytologic scorings after 18, 36 and 60 months of pill-taking are standardized and comparable to those used prior to the start of the trial.

F. References

1. Li JY, Liu BQ, Li GY, Chen ZJ, Sun XD, Rong SD: Atlas of Cancer Mortality in the People's Republic of China. Shanghai; China Map Press, 1979.

2. Esophageal Cancer Institute of Lin County: Lin County Cancer Registration. Unpublished results.

3. Munoz N, Crespi M, Grassi A, Wang GQ, Shen Q, Li ZC: Precursor lesions of esophageal cancer in high-risk populations in Iran and China. Lancet 1:876-9, 1982.

4. Li JY: The epidemiology of esophageal cancer in China. In Press.

5. Yang CS: Research on esophageal cancer in China: A review. Cancer Res 40:2633-44, 1980.

6. Coordinating Group for Research on the Etiology of Esophageal Cancer in North China. The epidemiology and etiology of esophageal cancer in North China: A preliminary report. Chin Med J 1:167-77, 1975.

7. Yang CS, Miao Y, Yang W, Huang M, Wang T, Xue H, You S, Lu J, Wu J: Diet and vitamin nutrition of the high esophageal cancer risk population in Linxian, China. Nutr Cancer 1982; 4(2):154-64.

8. O'Neill C, Pan QQ, Clarke G, Lin FS, Hodges G, Ge M, Jordan P, Chang YM, Newman R, Toulson E: Silica fragments from millet bran in mucosa surrounding esophageal tumors in patients in North China. Lancet 1:1202-6, 1982.

9. Li MH: Studies of potential carcinogens in the diet of individuals of high risk for esophageal cancer. In Cancer Research in the PRC and USA (ed. P.A. Marks). New York; Grune & Stratton, 131-6, 1981.

10. Lu SH, Camus AM, Tomatis L, Bartsch H: Mutagenicity of extracts of pickled vegetables collected in Linshien county, a high-incidence area for esophageal cancer in Northern China. J Natl Cancer Inst 66:33-6, 1981.

11. Mettlin C, Graham S, Priore R, Swanson M: Diet and cancer of the esophagus. Am J Epidemiol 112:422-3, 1980.

12. Ziegler RG, Morris LE, Blot WJ, Pottern LM, Hoover R, Fraumeni JF Jr: Esophageal cancer among black men in Washington, D.C. II. Role of nutrition. J Natl Cancer Inst 67:1199-206, 1981.

13. Wynder EL, Bross IJ: A study of etiological factors in cancer of the esophagus. Cancer 14:389-413, 1961.

14. Martinez I: Factors associated with cancer of the esophagus, mouth, and pharynx in Puerto Rico. J Natl Cancer Inst 42:1069-94, 1969.

15. Hirayama T: Diet and cancer. Nutr Cancer 1:67-81, 1979.

16. International Agency for Research in Cancer: Final report: Etiology of esophageal cancer in Caspian littoral of Iran. Lyon, IARC, 1981.

17. Van Rensburg SJ: Epidemiologic and dietary evidence for a specific nutritional predisposition to esophageal cancer. J Natl Cancer Inst 67:243-51, 1981.

18. Sporn MB, Dunlop NM, Newton DL, Smith JM: Prevention of chemical carcinogenesis by vitamin A and its synthetic analogs (retinoids). Fed Proc 35:1332-8, 1976.

19. Peto R, Doll R, Buckley JD, Sporn MB: Can dietary beta- carotene materially reduce human cancer rates. Nature 290:201-8, 1981.

20. Yang CS: Alterations in aryl hydorcarbon hydroxylase system during riboflavin depletion and repletion. Arch Biochem Biophys 160:623-30, 1974.

21. Foy H, Mbaya V: Riboflavin. Prog Food Nutr Sci 2:357-94, 1977.

22. Mirvish SS, Walleave L, Eagen M, Shubik P: Ascorbate- nitrite reaction: possible means of blocking the formation of carcinogenic N-nitroso compounds. Science 177:65-7, 1972.

23. Shklar G: Oral mucosal carcinogenesis in hamsters: inhibition by vitamin E. J Natl Cancer Inst 68:791-7. 1982.

24. Gabriel GN, Schranger TF, Newberne PM: Zinc deficiency, alcohol, and a retinoid association with esophageal cancer in rats. J Natl Cancer Inst 68:785-9, 1982.

25. Smith JC, McDaniel EG, Fan FF, Halstead JA: Zinc: a trace element essetial in vitamin A metabolism. Science 181:954, 1973.

26. Luo XM, Wei HJ, Yang SP: Inhibitory effects of molybdenum on esophageal and forestomach cancer in rats. J Natl Cancer Inst 71:75-80, 1983.

27. Recommended Daily Allowances, 9th edition, National Research Council, National Academy of Sciences, 1980, Washington, D.C.

28. Korner WF, Vollin J: New aspects of the tolerance of retinol in humans. Int J Vit Nutr Res 45:363-72, 1975.

29. Farrell PM, Bieri JG: Megavitamin supplementation in man. Am J Clin Nutr 28:1381, 1975.

30. Yang G, Wang S, Zhou R, Sun S: Endemic selenium intoxication of humans in China. Am J Clin Nutr 37:872-81, 1983.

31. Prasad AS: Deficiency of zinc in man and its toxicity. In: AS Prasad [ed.] Trace elements in human health and disease. Academic Press, New York, 1-20, 1976.

32. Deosthale YG, Gopalanc: The effect of molybdenum levels in sorghum (Sorgum Vulgare Pers) on uric acid and copper excretion in man. Br J Nutr 31:351-5, 1974.

33. Rothman KJ, Boice JD: Epidemiologic analysis with the programmable calculator. NIH Publication No. 79-1694, 1979.

34. Peto R, Pike MC, Armitage P, et al: Design and analysis of randomized clinical trials requiring prolonged observation of each patient. I. Introduction and design. Br J Cancer 34:585-612, 1976.

Appendix 1. Cytologic Diagnosis at Dysplasia

The balloon swallow technique was used to identify dysplasia subjects in previous mass screenings and will be used for subsequent periodic follow-up of persons enrolled in the dysplasia trial. The cytology slides will all be evaluated in China, under the direction of Professor Shen Qiong who pioneered this technique. Epithelial cells in the smear are classifed into five grades according to morphology, with emphasis on the structure of the nuclei.

Grade I - Normal: Cells in the smear in normal subjects are chiefly of intermediate type, 10-15% of superficial cells. Parabasal cells are rarely exfoliated, basal cells usually not.

Grade II - Hyperplasia: There is a mild degree of hyperchromasia of the cells. The chromatin content of the nucleus is increased, and the nucleus is 2 or more but less than 3 times greater in size than in normal cells of the same layer. The chromatin granules are fine and the nuclear membrane is not thickened.

Grade III - Marked severe dysplasia: The size of dysplastic nuclei of intermediate cells is three to five times that of the normal intermediate cells. Hyperchromasia is more marked. The chromatin granules become coarse but are rather uniform in size and even in distribution. The nuclear membrane is slightly thickened, but regular. Dysplastic parabasal cells are increased in number. To make a diagnosis, a total of five or more such cells is required. This type of dysplasia may be divided into two groups:

Group I. The nuclear size of dysplastic cells is 3 times or more that of normal cells in the same layer.

Group II. The nuclear size of dysplastic cells is 4 times or more that of normal cells in the same layer.

Grade IV - Near-carcinoma: The nuclei in the near-carcinoma cells are 5 or more times greater in size than those in the normal cells of the same layer. The chromatin granule of the nuclei are not as coarse as those of typical cancer cells. Near-carcinoma cells always lie in the epithelium adjacent to carcinoma in situ or in the epithelium overlying carcinoma of the basal layers.

Grade V - Early carcinoma: In this category typical cancer cells are presented in the smear. The diameter of the nucleus exceeds one third the diameter of the cell. The malignant feature of the cancer cell nuclei are very distinct. The chromatin granules are coarse, varied in size, and uneven in distribution. All these morphologic characteristics serve to distinguish carcinoma cells from near-carcinoma cells.

The subjects to be recruited for the dysplasia trial are those in Grade III - severe dysplasia.

Appendix 2: See tab under screener questionnaire

Appendix 3: See tab for study publications
