# Supplementary material for: Colorectal cancer in the Linxian China Nutrition Intervention Trial: Risk factors and intervention results
Source: PLoS One. 2021 Sep 15;16(9):e0255322. doi: 10.1371/journal.pone.0255322 (PMC8443060; doi:10.1371/journal.pone.0255322)
Supplement: S2 File — (DOCX) [file pone.0255322.s003.docx]

**Supplementary Table 1.** Distribution of selected baseline characteristics/exposures in the total cohort and by gender.

| **Characteristics** | **Total**  **Cohort**  **(n= 29,553)** | | **Gender Group** | | | |
| --- | --- | --- | --- | --- | --- | --- |
|  |  |  | **Male**  **(n=13,175)** | | **Female**  **(n=16,378)** | |
|  | **Cohort**  **%** | **Cases**  **%** | **Cohort**  **%** | **Cases**  **%** | **Cohort**  **%** | **Cases**  **%** |
| **Age** (years)  <50  ≥50 | 42  58 | 46  54 | 39  61 | 49  51 | 44  56 | 44  56 |
| **Gender**  Male  Female | 45  55 | 37  63 | - | - | - | - |
| **BMI** (kg/m^2^)^a^   - T _1_ - T _2_ - T _3_ | 33  33  34 | 31  29  40 | 27  39  34 | 25  36  39 | 32  34  34 | 28  30  42 |
| **Height** (m)^a^   - T _1_ - T _2_ - T _3_ | 33  35  32 | 27  42  31 | 30  39  31 | 22  42  36 | 34  30  36 | 25  29.5  45.5 |
| **Weight** (kg)^a^   - T _1_ - T _2_ - T _3_ | 33  34  33 | 28  37  35 | 26  41  33 | 19  42  39 | 36  34  30 | 24  38  38 |
| **Education**   - No formal education - 1-5 year - >5 years | 40  31  29 | 44  27  29 | 18  44  38 | 23  34  43 | 58  20  22 | 57  22  21 |
| **Born in Linxian** (Yes) | 96 | 96 | 97 | 95 | 95 | 96 |
| **Water piped into the home** (Yes) | 25 | 34 | 24.5 | 39 | 25 | 30 |
| **Alcohol intake** (Yes)^b^ | 23.5 | 26 | 40 | 45 | 10 | 14 |
| **Ever smoke** (Yes)^c^ | 30 | 23 | 67 | 61 | 0.2 | 0 |
| **Family history for any cancer** (Yes)^d^ | 34 | 33 | 35 | 31 | 34 | 34 |
| **Family history of colorectal cancer** (Yes)^d^ | 0.3 | 1.1 | 0.3 | 3 | 0.3 | 0 |

^a^BMI, height, and weight were divided into category-specific tertiles as follows. For the total cohort: BMI <20.81, 20.81-22.66, >22.66; Height <1.55, 1.55-1.62, >1.62; Weight <51.50, 51.50-58, >58. For males: BMI <20.46, 20.46-22.34, >22.34; Height <1.62, 1.62-1.67, >1.67; Weight <55, 55-61, >61. For females: BMI <20.81, 20.81-22.94, >22.94; Height <1.52, 1.52-1.55, >1.55; Weight <49, 49-55, >55.

^b^Alcohol intake was evaluated as any use in previous 12 months.

^c^Ever smokers were defined as those who had ever smoked regularly for at least 6 months.

^d^Family history of any cancer and of CRC are for cancer in 1^st^-degree relative(s). Note: total of 43 males and 50 females in the cohort reported family history of CRC.

**Supplementary Table 2.** Distribution of food consumption in the total cohort and by gender.

| **Food^a^** | **Total**  **Cohort**  **(n= 29,553)** | | **Gender Group** | | | |
| --- | --- | --- | --- | --- | --- | --- |
|  |  |  | **Male**  **(n=13,175 )** | | **Female**  **(n=16,378 )** | |
|  | **Cohort**  **%** | **Cases**  **%** | **Cohort**  **%** | **Cases**  **%** | **Cohort**  **%** | **Cases**  **%** |
| Foods cooked in oil   - T_1_ - T_2_ - T_3_ | 32  36  32 | 22  42  36 | 35  29  36 | 37  29  34 | 36  36  28 | 26  37  37 |
| Meat   - T_1_ - T_2_ - T_3_ | 33  52  15 | 30  49  21 | 37  41  22 | 24  42  34 | 29  38  33 | 33  29  38 |
| Eggs   - T_1_ - T_2_ - T_3_ | 28  46  26 | 23  42  35 | 36  35  29 | 24  30  46 | 32  36  32 | 27  36  37 |
| Fresh fruits   - T_1_ - T_2_ - T_3_ | 34  30  36 | 32  24  44 | 32  32  36 | 21  22  57 | 34  39  27 | 33  37.5  29.5 |
| Fresh vegetables   - T_1_ - T_2_ - T_3_ | 32  29  39 | 31  27  42 | 31  28  41 | 30  30  40 | 33  29  38 | 31  26  43 |
| Pickled vegetables   - 0 - ≥1 | 91  9 | 91  9 | 92  8 | 99  1 | 89  11 | 87  13 |
| Moldy vegetables   - 0 - ≥1 | 99  1 | 100  0 | 99  1 | 100  0 | 99  1 | 100  0 |
| Millet chaff/persimmon bread   - 0 - ≥1 | 95  5 | 96  4 | 95  5 | 97  3 | 95  5 | 96  4 |
| Moldy bread   - 0 - ≥1 | 82  18 | 83  17 | 84  16 | 87  13 | 80  20 | 81  19 |
| Dried food (veg+fruit)   - <78 - ≥78 | 49  51 | 50  50 | 51  49 | 51  49 | 49  51 | 51  49 |

^a^Foods cooked in oil, meat, eggs, fresh vegetables, and fresh fruit consumption were divided into category-specific tertiles as follows. For the total cohort: Foods cooked in oil <8, 8-12, >12 servings/year; meat <6, 6-12, >12 servings/year; eggs <4, 4-24, >24 servings/year; fresh fruits <6, 6-23, >23 servings/year; fresh vegetables <730, 730-912, >912 servings/year. For males: Foods cooked in oil <10, 10-20, >20 servings/year; meat <8, 8-12, >12 servings/year; eggs <5, 5-24, >24 servings/year; fresh fruits <5, 5-16, >16 servings/year; fresh vegetables <549, 549-731, >732 servings/year. For females: Foods cooked in oil <8, 8-12, >12 servings/year; meat <5, 5-10, >10 servings/year; eggs <4, 4-12, >12 servings/year; fresh fruits <4, 4-17, >17 servings/year; fresh vegetables <549, 549-730, >730 servings/year.

**Supplementary Table 3.** Multivariate-adjusted RRs and 95% CIs for colon and rectal cancer cases for selected characteristics/exposures in the total cohort.^a^

|  | **Total Cohort**  (n=29,553) | | |
| --- | --- | --- | --- |
|  |  | **Colon** | **Rectum** |
| **Characteristics/exposures** | **No.C/R** | **RR (95% CI; p-value)** | **RR (95% CI; p-value)** |
| **Age (year)^b^** | 63/116 | **1.05 (1.01-1.09;0.008)** | 1.021 (0.995-1.047;0.108) |
| **Gender(male)^b^** | 18/49 | 0.48 (0.18-1.26) | 0.97 (0.51-1.85) |
| **BMI (kg/m^2^)^b,c^**   - T_1_ - T_2_ - T_3_ | 22/33  19/34  22/49 | 1  0.85 (0.46-1.58)  0.92 (0.51-1.67)  P_Trend_=0.812 | 1  0.96 (0.59-1.56)  1.36 (0.87-2.13)  P_Trend_=0.130 |
| **BMI^Chinese^ (kg/m^2^)^b,d^**   - <18.50 - 18.50-23.99 - ≥24.0 | 3/3  49/90  11/23 | 1  1.25 (0.39-4.03)  1.14 (0.32-4.12)  P_Trend_=0.360 | 1  1.97 (0.62-6.24)  2.21 (0.66-7.38)  **P_Trend_=0.023** |
| **Height (m)^b,c^**   - T_1_ - T_2_ - T_3_ | 17/32  32/43  14/41 | 1  **2.17 (1.18-3.97;0.013)**  1.73 (0.70-4.31)  P_Trend_=0.097 | 1  1.36 (0.84-2.20)  1.76 (0.94-3.32)  P_Trend_=0.076 |
| **Weight (kg)^b,c^**   - T_1_ - T_2_ - T_3_ | 22/28  22/45  19/43 | 1  1.14 (0.62-2.10)  1.17 (0.59-2.29)  P_Trend_=0.842 | 1  1.58 (0.97-2.58)  1.59 (0.94-2.69)  P_Trend_=0.103 |
| **Education^b^**   - No formal education - 1-5 year - >5 years | 31/48  22/48  10/20 | 1  0.79 (0.41-1.53)  0.69 (0.32-1.51) | 1  0.82 (0.51-1.33)  0.72 (0.40-1.28) |
| **Born in Linxian (yes)^b^** | 61/111 | 1.45 (0.35-5.96) | 1.00 (0.40-2.45) |
| **Water piped into home (yes)^b^** | 20/40 | 1.38 (0.81-2.35 | **1.56 (1.06-2.30;0.022)** |
| **Alcohol intake (yes)^b,e^** | 16/30 | 1.50 (0.81-2.80) | 1.12 (0.71-1.76) |
| **Ever smoke (yes)^b,f^** | 11/30 | 0.76 (0.29-1.96) | 0.81 (0.45-1.45) |
| **Family history of any cancer (yes)^b,g^** | 20/39 | 0.92 (0.54-1.56) | 0.96 (0.70-1.31) |

^a^Multivariate-adjusted Cox proportional hazards regression models were used to estimate all RRs and 95% CIs shown. P-values are provided for all RRs whenever the p-value is less than 0.05 and for all P_trend_ tests. Bolded RRs, P_trend_ tests, and P-interaction tests indicate P<0.05.

^b^RRs and 95% CIs shown came from a single multivariate model for each cancer site (ie, Colon or Rectum) that included all 12 characteristic variables (only one BMI variable was used at a time). Body size variables were coded as BMI (divided into tertiles as T1,T2,T3 represented by indicator variables) or BMI^Chinese^ (divided into China-specific categories as <18.50, 18.50-23.99, ≥24.0 represented by indicator variables); Height (divided into tertiles as T1,T2,T3 represented by indicator variables); and Weight (divided into tertiles as T1,T2,T3 represented by indicator variables) where the first tertile was the reference category for each variable. The non-body size variables included in the multivariate model were Age (continuous), Gender (0=F,1=M), Education (0=No formal education, 1=1-5 years, and 2=>5 years represented by indicator variables), Born in Linxian (0=no,1=yes), Water piped into home (0=no,1=yes), Alcohol intake (0=no,1=yes), Ever smoke (0=no,1=yes), and Family history of any cancer (0=no,1=yes).

P_trend_ tests (for the ordinal variables of BMI, BMI^Chinese^, Height, Weight, and Education) were evaluated as a single ordinal variable (0,1,2) to account for the three categories of each variable as shown and ordered in the table.

**Further description of variables:** ^c^BMI, height, and weight were divided into category-specific tertiles as follows: BMI <20.81, 20.81-22.66, >22.66; Height <1.55, 1.55-1.62, >1.62; Weight <51.50, 51.50-58, >58. ^d^BMI was also divided into Chinese-specific BMI categories. ^e^Alcohol intake was evaluated as any in previous 12 months. ^f^Ever smokers were defined as those who had ever smoked regularly for at least 6 months. ^g^Family history of any cancer in 1^st^degree relative(s).

**Supplementary Table 4.** Multivariate-adjusted RRs and 95% CIs for colon and rectal cancer cases by consumption of selected foods in the total cohort.^a^

|  | **Total Cohort** (n=29,553) | | |
| --- | --- | --- | --- |
| **Consumption frequency**  **(Times/year)** | No.  C/R | **Colon** | **Rectum** |
|  |  | **RR (95% CI; p-value)** | **RR (95% CI; p-value)** |
| **Foods cooked in oil^b,c^**   - T_1_ - T_2_ - T_3_ | 19/21  24/51  20/44 | 1  1.11 (0.60-2.03)  1.07 (0.57-2.03)  P_Trend_=0.916 | **1**  **2.03 (1.22-3.40;0.007)**  **2.02 (1.19-3.41;0.009)**  P_Trend_=0.089 |
| **Meat^b,c^**   - T_1_ - T_2_ - T_3_ | 15/39  36/52  12/25 | 1  1.57 (0.86-2.89)  2.05 (0.93-4.52)  P_Trend_=0.117 | 1  0.80 (0.52-1.21)  1.30 (0.77-2.21)  P_Trend_=0.160 |
| **Eggs^b,c^**   - T_1_ - T_2_ - T_3_ | 14/27  24/51  25/38 | 1  1.06 (0.55-2.06)  **1.99 (1.02-3.88;0.044)**  **P_Trend_=0.013** | 1  1.11 (0.70-1.78)  1.48 (0.89-2.44)  P_Trend_=0.107 |
| **Fresh fruits^b,c^**   - T_1_ - T_2_ - T_3_ | 21/43  17/37  25/36 | 1  0.99 (0.52-1.89)  **1.85 (1.02-3.37;0.043)**  **P_Trend_=0.018** | 1  0.97 (0.62-1.52)  1.13 (0.72-1.79)  P_Trend_=0.974 |
| **Fresh vegetables^b,c^**   - T_1_ - T_2_ - T_3_ | 18/38  19/29  5/14 | 1  1.17 (0.61-2.23)  0.81 (0.30-2.20)  P_Trend_=0.812 | 1  0.87 (0.54-1.42)  1.11 (0.60-2.06)  P_Trend_=0.866 |
| **Pickled vegetables^b^**   - 0 - ≥1 | 55/108  8/8 | 1  1.33 (0.63-2.79) | 1  0.70 (0.34-1.44) |
| **Millet chaff/Persimmon bread^b^**   - 0 - ≥1 | 60/112  3/4 | 1  1.10 (0.34-3.53) | 1  0.81 (0.30-2.21) |
| **Moldy bread^b^**   - 0 - ≥1 | 51/98  12/18 | 1  1.03 (0.55-1.93) | 1  0.99 (0.68-1.46) |
| **Dried food (veg/fruit)^b^**   - <78 - ≥78 | 33/57  30/59 | 1  0.94 (0.56-1.58) | 1  1.14 (0.78-1.67) |

^a^Separate multivariate-adjusted Cox proportional hazards regression models were used to estimate RRs and 95% CIs for each of the 9 different analyses shown here. Foods cooked in oil, Meat, Eggs, Fresh fruits, and Fresh vegetables were divided into tertiles as T1,T2,T3 represented by indicator variables with the first tertile serving as the reference category for each variable. Ptrend tests for these ordinal variables were evaluated as a single ordinal variable (0,1,2) to account for the three categories of each variable as shown and ordered in the table. P-values are provided for all RRs whenever the p-value is less than 0.05 and for all Ptrend tests. Bolded RRs and Ptrend tests indicate P<0.05.

^b^Multivariate models were adjusted for: Age (continuous), Gender (0,1), BMI (continuous), Education (0,1,2), Born in Linxian (0=No,1=Yes), Water piped into home (0=No,1=Yes), Alcohol intake (0=No, 1=Yes), Smoking (0=No, 1=Yes), and Family history of any cancer (0=No, 1=Yes). For more details regarding coding of these variables, see footnotes to Table 1.

^c^Further description of consumption frequency variables (times/year) divided into tertiles in the table: Foods cooked in oil, meat, eggs, fresh vegetables, and fresh fruits consumption were divided into category-specific tertiles as follows: Foods cooked in oil <8, 8-12, >12; meat<6, 6-12, >12; eggs <4, 4-24, >24; fresh fruits <6, 6-23, >23; fresh vegetables <730, 730-912, >912.

**Supplementary Table 5.** RRs and 95% CIs for colon and rectum by intervention group in all trial participants.

|  |  | | **Anatomic site** | | | |
| --- | --- | --- | --- | --- | --- | --- |
|  | **All**  **(n=179 CRCs)^a^** | | **Colon**  **(n=63)^a^** | | **Rectum**  **(n=116)^a^** | |
|  | **RR (95% CI)** | **P** | **RR (95% CI)** | **P** | **RR (95% CI)** | **P** |
| **Factor A^b^** | 1.07 (0.80-1.43) | 0.659 | 1.33 (0.81-2.19) | 0.262 | 0.94 (0.66-1.36) | 0.760 |
| **Factor B^b^** | 0.79 (0.59-1.06) | 0.118 | 0.67 (0.40-1.10) | 0.116 | 0.87 (0.60-1.25) | 0.459 |
| **Factor C^b^** | 1.07 (0.80-1.43) | 0.641 | 1.49 (0.90-2.47) | 0.122 | 0.89 (0.62-1.29) | 0.551 |
| **Factor D^b^** | 0.77 (0.57-1.03) | 0.081 | 0.67 (0.40-1.10) | 0.115 | 0.83 (0.58-1.20) | 0.334 |

^a^Separate multivariate Cox proportional hazards regression models were used to estimate RRs and 95% CIs for All, Colon, and Rectum. These models included adjustment for the 3 factors that trial participants were blocked on at randomization (ie, commune, age, and gender) and 4 separate indicator variables which represented each of the 4 intervention treatment groups evaluated in the model (Factor A=0 if not A, 1 if A; Factor B=0 if not B, 1 if B; Factor C=0 if not C, 1 if C; and Factor D=0 if not D, 1 if D).

^b^Micronutrients included in the 4 intervention treatments were: Factor A (5000 IU vitamin A and 22.5 mg zinc oxide), factor B (3.2 mg riboflavin and 40 mg niacin), factor C (120 mg ascorbic acid and 30 ug molybdenum), and factor D (50 ug selenium, 30 mg alpha-tocopherol, and 15 mg beta-carotene).

**Supplementary Table 6:** Summary of colorectal cancer risk factors in the West and in prospective cohort studies of Asian Chinese.

|  | **Prospective Cohort Studies of CRC in Asian Chinese^a,b,c^** | | | | | |
| --- | --- | --- | --- | --- | --- | --- |
|  | **Rural Chinese cohorts** | | **Urban Chinese cohorts** | | | |
| **Western CRC risk factors** | **NIT^d^** | **Jiashan** | **SCS** | **Singapore** | **SMHS** | **SWHS** |
| **NON-DIET RELATED** |  |  |  |  |  |  |
| ↑ age | **↑RR=1.03/y (all,F);1.04/y (M)** |  |  |  |  | ↑RR=3.8 (>65 vs <45y) |
| Male gender | Null |  |  |  |  |  |
| Family history of CRC | **↑RR=9.53 (M)** |  |  |  |  | Null |
| Smoking | Null (M) | Null | Null^e^ | ↑RR=1.49 | ↑RR=1.20 |  |
| Education | Null |  |  |  |  | Null |
| **DIET-RELATED** |  |  |  |  |  |  |
| ↑ body fatness | Null for BMI^tertiles^; BMI^Chinese^ ↑RR=1.68**, ptrend=0.017 (all);** ↑RR=2.06**, ptrend=0.002 (F)** |  | ↑BMI^f^ | ↑RR=1.25 (BMI) | ↑RR=2.15 (BMI);RR=1.97 (WHR) | Null for BMI,WHR,WC |
| ↑ weight | Null |  |  |  |  |  |
| ↑ height | **↑RR=1.80, ptrend=0.016 (all);** ↑RR=1.83**, ptrend=0.014 (F)** |  |  |  |  |  |
| ↓ physical activity |  |  |  | ↓RR=0.61 | Null | Null |
| **DIET** |  |  |  |  |  |  |
| Alcohol intake | Null | Null (all,M,F) |  | ↑RR=1.84 | ↑RR=1.33 |  |
| ↑ processed meat, red meat, meat | ↑RR=1.51, **ptrend=0.04 (all);** ↑RR=1.61**, ptrend=0.046 (M)** | Null |  | Null,null | Null | Null (red meat,total meat) |
| ↓ whole grains,fiber,dairy,Ca^++^ |  |  |  | Null(fiber),null(Ca++) |  | ↓ptrend=0.05 (milk; colon) |
| ↓ fish |  | Null |  | Null |  | Null |
| ↓ vegetables | Null | Null |  | Null | Null | Null |
| ↓ fruit | **↑RR=1.97, ptrend=0.004 (M***)* |  |  | Null | ↓RR=0.67 | Null |
| Pickled foods | Null |  |  |  |  |  |
| Fried foods | Null |  |  |  |  |  |
| **OTHER DIET FACTORS** |  |  |  |  |  |  |
| Eggs | **↑RR=1.65, ptrend=0.005 (all); 2.14, ptrend=0.004 (M)** |  |  |  |  | ↑RR=1.4 |
| Green tea |  |  |  | ↑RR=1.31 (M) | ↓RR=0.54 (nonsmokers) | ↓RR=0.63 |
| Black tea |  |  |  | Null |  |  |
| Coffee |  |  |  | Null |  |  |
| Eel,shrimp,shellfish |  |  |  |  |  | ↑RR=1.3 (for eel,shrimp,shellfish) |
| Cholesterol |  |  |  |  |  | ↑RR=1.6 |
| Milk |  |  |  |  |  | ↓ptrend=0.05 (milk; colon) |
| Fats (total,SFA,MUFA,PUFA) |  |  |  | Null |  | Null |
| Marine n-3 PUFA |  |  |  | ↑RR=1.21 |  |  |
| Foods cooked in oil | **↑RR=1.57 (all); ↑RR=1.70 (F)** |  |  |  |  | Null (deep frying,stir frying) |
| Smoked cooking |  |  |  |  |  | ↑RR=1.4 (colon) |
| Watermelon |  |  |  |  | ↓RR=0.77 |  |
| **THE OTHER FACTORS** |  |  |  |  |  |  |
| Water source | **↑RR=1.50 (all); ↑RR=1.94 (M)** (piped into home) | ↑RR=2.02 (all) (well water) |  |  |  |  |
|  |  |  |  |  |  |  |

^a^Null=association P>0.05; M=male, F=female; C=colon only; RRs shown have a quartile or p-trend with P<0.05.

^b^Study abbreviations: NIT=Nutrition Intervention Trial;SCS=Shanghai Cohort Study;Singapore=Singapore Chinese Health Study;SMHS=Shanghai Mens' Health Study;SWHS=Shanghai Womens' Health Study.

^c^Other abbreviations: CRC=colorectal cancer; ASA=aspirin; HRT=hormone replacement therapy; Ca^++^=calcium, SFA=saturated fatty acids; MUFA=monounsaturated fatty acids; PUFA=polyunsaturated fatty acids; RR=relative risk; BMI=body mass index; WHR=waist hip ratio; WC=waist circumference.

^d^The factors/results shown in **bold** are nominally significant in the Linxian NIT findings (P<0.05). Empty cell indicates lack of data for a specific factor/result.

^e^The SCS smoking results were based on small numbers of CRCs ascertained as of 1996 (26 colon & 31 rectal cancers).

^f^The SCS report on BMI was published in Chinese, with only an abstract in English; CRC deaths reportedly increased with BMI, but specifics not given.

**Supplementary Table 7.** Micronutrient trials and colorectal adenoma (CRA) prevention.^a^

| **RCT no.** | **RCT name** | **1st Author** | **Citation/Reference** | **Population** | **Age**  **(mean±SD)** | **Intervention** | **Duration** | **No. CRA endpoints^b^** | **Results^c^**  **(RR, OR or HR (95% CI))** |
| --- | --- | --- | --- | --- | --- | --- | --- | --- | --- |
| 1 | Ca Polyp Prevention Trial | Baron JA. | N Engl J Med 1999;340:101-7. | 930 M/F (USA) | 61±9y | Ca (1200mg/d) | 3y | 286 (of 832) | ***RR=0.81(0.67-0.99)*** |
| 1 | Ca Follow-up Study | Grau MV. | J Natl Cancer Inst 2007;99:129-36. | Observ. phase 1 (1-5y post-tx) |  |  | 10y | 131 (of 347) | ***RR=0.63(0.46-0.87)(1-5y post-tx)*** |
|  |  |  |  | Observ. phase 2 (6-10y post-tx) |  |  |  | 164 (of 424) | RR=1.09(0.85-1.39)(6-10y post-tx) |
| 2 | ECP Intervention Study | Bonithon-Kopp C. | Lancet 2000;356:1300-6. | 665 M/F (Europe,10 countries) | 59.1±8.7y | Ca (2000mg/d) | 3y | 122 (of 552) | OR=0.66(0.38-1.17) |
| 3 | ASA/Folate Polyp Prevention Trial | Cole BF. | JAMA 2007;297:2351-9. | 1021 M/F (USA) | 57±9.6y | Folate (1mg/d) | 3y | 427 (of 987) | HR=1.04(0.90-1.20) |
|  |  |  |  |  |  |  | 6y |  | HR=1.13(0.93-1.37) |
| 4 | ukCAP | Logan RF. | Gastroenterology 2008;134:29-38. | 945 M/F (UK, DK) | 57.8±9y | Folate (0.5mg/d) | 3y | 220 (of 853) | HR=1.07(0.85-1.34) |
| 5 | Colorectal Chemoprev Pilot Study (SWOG-9041) | Chu DZ. | Clin Colorectal Ca 2011;10:310-6. | 220 M/F resected CRC pts (USA,Canada) | 68y (median) | Ca (1800mg/d) | 5y | 104 (of 194) | ***OR=0.47(0.27-0.84)*** |
| 6 | WAFACS | Song Y. | J Natl Cancer Inst  2012;104:1562-75. | 1470 F (USA) | 62.1±7.6y | Folate (2.5mg/d) +  B6 (50mg/d) +  B12 (1mg/d) | 9.2y (7.3y tx+1.9y post-tx) | 355 (of 1470) | HR=1.00(0.83-1.20) |
| 7 | Vitamin D/Ca Polyp Prevention Trial | Baron JA. | N Engl J Med 2015;373:1519-30. | 2259 M/F (USA) | 58.1±6.8y | Vit D (1000IU/d) | 3 or 5y | 880 (of 2059) | RR=0.99(0.89-1.09)^d^ |
|  |  |  |  |  |  | Ca (1200mg/d) |  | 707 (of 1523) | RR=0.95(0.85-1.06)^d^ |
| 7 | Vitamin D/Ca Polyp Prevention Trial | Crockett SD. | Gut 2018;Mar 1.pii: gutjnl-2017-315242. | 2259 M/F (USA) |  | Vit D (1000IU/d) | 3 or 5y | 565 (of 2058) | RR=1.01(0.87-1.17)^e^ |
|  |  |  |  |  |  | Ca (1200mg/d) |  |  | RR=1.15(0.98-1.36)^e^ |
|  |  |  |  | Observation phase (3-5y post-tx) |  |  | 4.6y post tx (aver) | 329 (of 1108) | Vit D: RR=1.04(0.86-1.25)^e^ |
|  |  |  |  |  |  |  |  |  | Ca: RR=1.21(0.97-1.50)^e^ |
|  |  |  |  |  |  |  |  | 62 (of 1108) | ***Ca:RR=2.66(1.44-4.89)^f^*** |
| 7 | Vitamin D/Ca Polyp Prevention Trial | Calderwood AH. | Ca Prev Res 2019;12:295-304. | Observation phase (3-5y post-tx) |  |  | 4.6y post tx (aver) | 569 (of 1121) | Vit D: RR=1.04(0.93-1.17)^g^ |
|  |  |  |  |  |  |  |  |  | Ca: RR=0.95(0.84-1.08)^g^ |
| 8 | Se & Celecoxib Trial (Sel-Cel) | Thompson PA. | J Natl Cancer Inst 2016; 108(12). pii: djw152. | 1621 M/F (USA) | 63.2±9.0y | Se (200ug/d) | 3y (median) | 597 (of 1374) | RR=1.03(0.91-1.16) |
| 9 | SELECT (ancillary study) | Lance P. | Ca Prev Res 2016;10:45-54. | 8094 M (USA, Canada, PR) | 62.0y | Se (200ug/d) | 5.46y (median) | 2286 (of 6546) | RR=0.96(0.90-1.02) |
|  |  |  |  |  |  | AT (400IU/d) |  |  | RR=1.03(0.96-1.10) |

^a^Abbreviations: Population (PR=Puerto Rico); Intervention (Ca=calcium, Se=selenium, AT=alpha-tocopherol); Results (RR=relative risk, OR=odds ratio, HR=hazards ratio), CI=confidence interval.

^b^Number of CRA endpoints (number of evaluable patients).

^c^Bold and italicized results indicate relative risk confidence intervals that do not overlap 1.00 and are p<0.05.

^d^Results from analysis of effect of vitamin D and Ca supplementation on all polyps in treatment phase.

^e^Results from subanalysis of effect of vitamin D and Ca supplementation on all serrated polyps in observational phase.

^f^Results from sub- subgroup analysis of effect of Ca supplementation on sessile serrated adenomas or polyps (SSA/P, approx 12% of all serrated polps in this trial) in observational phase.

^g^Results from analysis of effect of vitamin D and Ca supplementation on all polyps in observational phase.

**Supplementary Table 8.** Micronutrient trials and colorectal cancer (CRC) prevention.^a,b^

| **RCT no.** | **RCT name** | **1st Author** | **Citation/Reference** | **Population** | **Age (mean±SD)** | **Intervention** | **Duration** | **No. CRC endpoints** | **Results^b^**  **(RR, HR (95% CI))** |
| --- | --- | --- | --- | --- | --- | --- | --- | --- | --- |
| 1 | ATBC | ATBC Study Grp Investigators | N Engl J Med 1994; 330:1029-35. | 29,133 M (Finland) | 57.2y | AT (50mg/d) | 6y (median) | 149 | AT/no AT case ratio=68/81=0.92 |
|  |  |  |  |  |  | BC (20mg/d) |  |  | BC/no BC case ratio=76/73=1.04 |
| 1 | ATBC | Virtamo J. | JAMA 2003;290:476-85. | 25,565 M (Finland) |  |  | Trial (1986-93) | 135 | AT: RR=0.78(0.55-1.09) |
|  |  |  |  |  |  |  |  |  | BC: RR=1.05(0.75-1.47) |
|  |  |  |  |  |  |  | Post-trial 1 FU (1993-6) | 92 | AT: RR-1.19(0.79-1.80) |
|  |  |  |  |  |  |  |  |  | BC: RR=1.06(0.70-1.60) |
|  |  |  |  |  |  |  | Post-trial 2 FU (1996-9) | 113 | AT: RR=1.02(0.70-1.47) |
|  |  |  |  |  |  |  |  |  | ***BC: RR=1.88(1.28-2.76)*** |
| 2 | PHS | Hennekens CH. | N Engl J Med 1996; 334:1145-49. | 22,071 M (USA) | 40-84y | BC (50mg qod) | 12y (average) | 341 | BC/no BC case ratio=167/174=0.96 |
| 2 | PHS | Cook NR. | Cancer Caus Cont 2000; 11:617-26. |  |  |  |  | 267 (colon) | RR=.9(0.7-1.2) |
|  |  |  |  |  |  |  |  | 79 (rectum) | RR=1.1(0.7-1.8) |
| 3 | CARET | Omenn GS. | J Natl Cancer Inst 1996; 88:1550-59. | 18,314 M (USA) | 58y | BC (30mg/d) + Vit A (25000IU/d) | 4y (average) | 106 | RR=1.02(0.70-1.50) |
| 4 | NPC | Clark LC. | JAMA 1996;276:1957-63. | 1312 M/F (USA) | 63.2±10y | Se (200ug/d) | 4.5y Tx + 6.4y FU (average) | 27 | ***RR=0.42(0.18-0.95)*** |
| 4 | NPC | Duffield-Lillico AJ. | Cancer Epidemiol Biomarkers Prev 2002;11:630-9. |  |  |  | 4.5y Tx + 7.4y FU (average) | 28 | HR=0.46(0.21-1.02) |
| 5 | HOPE & HOPE-TOO | Lonn E. | JAMA 2005;293:1338-47. | 9541 M/F  4732 (3994 Tx,738 FU only) (USA, Canada, Mexico, S.America, Europe) | 66±7y | AT (400IU/d) | 4.5y Tx2.6y Tx  and/or FU  Overall FU 7y (median) | 126 | RR=1.22(0.86-1.73) |
| 6 | WHS | Lee IM. | JAMA 2005;294:56-65. | 38,876 F (USA) | 54.6±7.0y | AT (600IU qod) | 10y (average) | 214 (colon) | RR=1.00(0.77-1.31) |
| 7 | WHI (CaD) | Wactawski-Wende J. | N Engl J Med 2006; 354:684-96. | 36,282 F (USA) | 50-79y | Ca (1gm/d) + Vit D (400IU/d) | 7y (average) | 322 | HR=1.08(0.86-1.34) |
| 7 | WHI (CaD) | Brunner RL. | Nutr Cancer 2011; 63:827-41. |  |  |  | 7y (average) | 235 (colon) | HR=0.98(0.76-1.27) |
|  |  |  |  |  |  |  |  | 70 (rectum) | HR=1.42(0.88-2.28) |
| 8 | HOPE-2 | Lonn E. | N Engl J Med 2006; 354:1567-77. | 5522 M/F (USA, Canada, Brazil, W Europe, Slovakia) | 68.8±7y | Folate (2.5mg/d) + B6 (50mg/d) + B12 (1mg/d) | 5y (average) | 87 (colon) | RR=1.36(0.89-2.08) |
| 9 | WAFACS | Zhang SM. | JAMA 2008;300:2012-21. | 5442 F (USA) | 62.8±8.8y | Folate (2.5mg/d) + B6 (50mg/d) + B12 (1mg/d) | 7.3y | 40 | HR=0.81(0.43-1.50) |
| 10 | WACS | Lin J. | J Natl Cancer Inst 2009; | 7627 F (USA) | 60.4±9y | Vit C (500mg/d) | 9.4y (average) | 44 | RR=0.76(0.42-1.38) |
|  |  |  | 101:14-23. |  |  | AT (600IU qod) |  |  | RR=0.63(0.34-1.15) |
|  |  |  |  |  |  | BC (50mg qod) |  |  | RR=1.32(0.73-2.39) |
| 11 | SELECT | Lippman SM. | JAMA 2009;301:39-51. | 35,533 M (USA, Canada,PR) | 62.6y (median) | Se (200ug/d)[Se vs Placebo] | 5.46y (median) | 123 | HR=1.09(0.69-1.73) |
|  |  |  |  |  |  | AT (400IU/d)[AT vs Placebo] |  | 126 | HR=1.05(0.66-1.67) |
| 11 | SELECT | Klein EA. | JAMA 2011;306:1549-56. |  |  | Trial + Post-trial FU (Se) | 7y (minimum) | 149 | HR=0.96(0.63-1.46) |
| 12 | PHS II | Gaziano JM. | JAMA 2009;301:52-62. | 14,641 M (USA) | 64.2±9y | AT (400IU qod) | 8y (average) | 162 | HR=0.88(0.64-1.19) |
|  |  |  |  |  |  | Vit C (500mg/d) |  |  | HR=0.86(0.63-1.17) |
| 12 | PHS II | Gaziano JM. | JAMA 2012;308:1871-80. |  |  | MV (daily) | 11.2y (median) | 210 | HR=0.89(0.68-1.17) |
| 12 | PHS II | Wang L. | Am J Clin Nutr 2014; 100:915-23. |  |  | Trial + Post-trial FU (AT) | 7.6y Tx + 2.8y FU (average) | 210 | HR=0.92(0.70-1.21) |
|  |  |  |  |  |  | Trial + Post-trial FU (Vit C) |  | 210 | HR=0.79(0.61-1.04) |
| 13 | NORVIT & | Ebbing M. | JAMA 2009;302:2119-26. | 6837 M/F (Norway) | 62.3±11y | Folate (.8mg/d)) + B6 (40mg/d) + B12(.4mg/d) | 3.3y Tx (median) | 95 | HR=1.00(0.59-1.69) |
|  | WENBIT |  |  |  |  |  | 3.2y FU (median) |  | HR=1.18(0.69-2.00) |
| 14 | VITAL | Manson JE. | N Engl J Med 2019; 380:33-44. | 25,871 M/F (USA) | 67.1±7.1y | Vit D (2000IU/d) | 5.3y (median) | 98 | HR=1.09(0.73-1.62) |

^a^Abbreviations: Population (PR=Puerto Rico); Intervention (AT=alpha-tocopherol,BC=beta-carotene, Se=selenium, Ca=calcium,MV=multivitamin); Duration (Tx=treatment, FU=followup); Results (RR=relative risk, HR=hazard ratio), CI=confidence interval.

^b^Bold indicates relative risk with confidence interval that does not include 1.00 and p<0.05.
